# ASXL3 bridges BRD4 to BAP1 complex and governs enhancer activity in small cell lung cancer

**DOI:** 10.1186/s13073-020-00760-3

**Published:** 2020-07-15

**Authors:** Aileen Patricia Szczepanski, Zibo Zhao, Tori Sosnowski, Young Ah Goo, Elizabeth Thomas Bartom, Lu Wang

**Affiliations:** 1 Simpson Querrey Center for Epigenetics, Northwestern University Feinberg School of Medicine, 303 East Superior Street, Chicago, IL 60611 USA; 2 Department of Biochemistry and Molecular Genetics, Northwestern University Feinberg School of Medicine, 303 East Superior Street, Chicago, IL 60611 USA; 3 Proteomics Center of Excellence, Northwestern University, 2145 North Sheridan Rd, Evanston, IL 60208 USA

**Keywords:** ASXL3, BAP1 complex, BRD4, SCLC, Enhancer activity, BET inhibitors

## Abstract

**Background:**

Small cell lung cancer (SCLC) is a more aggressive subtype of lung cancer that often results in rapid tumor growth, early metastasis, and acquired therapeutic resistance. Consequently, such phenotypical characteristics of SCLC set limitations on viable procedural options, making it difficult to develop both screenings and effective treatments. In this study, we examine a novel mechanistic insight in SCLC cells that could potentially provide a more sensitive therapeutic alternative for SCLC patients.

**Methods:**

Biochemistry studies, including size exclusion chromatography, mass spectrometry, and western blot analysis, were conducted to determine the protein-protein interaction between additional sex combs-like protein 3 (ASXL3) and bromodomain-containing protein 4 (BRD4). Genomic studies, including chromatin immunoprecipitation sequencing (ChIP-seq), RNA sequencing, and genome-wide analysis, were performed in both human and mouse SCLC cells to determine the dynamic relationship between BRD4/ASXL3/BAP1 epigenetic axis in chromatin binding and its effects on transcriptional activity.

**Results:**

We report a critical link between BAP1 complex and BRD4, which is bridged by the physical interaction between ASXL3 and BRD4 in an SCLC subtype (SCLC-A), which expresses a high level of *ASCL1*. We further showed that ASXL3 functions as an adaptor protein, which directly interacts with BRD4’s extra-terminal (ET) domain via a novel BRD4 binding motif (BBM), and maintains chromatin occupancy of BRD4 to active enhancers. Genetic depletion of ASXL3 results in a genome-wide reduction of histone H3K27Ac levels and BRD4-dependent gene expression in SCLC. Pharmacologically induced inhibition with BET-specific chemical degrader (dBET6) selectively inhibits cell proliferation of a subtype of SCLC that is characterized with high expression of ASXL3.

**Conclusions:**

Collectively, this study provides a mechanistic insight into the oncogenic function of BRD4/ASXL3/BAP1 epigenetic axis at active chromatin enhancers in SCLC-A subtype, as well as a potential new therapeutic option that could become more effective in treating SCLC patients with a biomarker of ASXL3-highly expressed SCLC cells.

## Background

Recent advances have shown that epigenetics, which represents heritable changes in gene expression that occur independent from changes in the primary DNA sequence, plays an important role in human cancer development [1]. Dysregulations and mutations within epigenetic factors, such as histone lysine methyltransferases [2–5], deubiquitinases [6], DNA methyltransferases [7], and DNA demethylases [8], are common mechanisms driving tumorigenesis. Therefore, identification of new epigenetic biomarkers and/or targets may contribute to the development of novel cancer therapeutics.

The BRCA1-associated protein 1 (BAP1) complex was initially identified as a major histone H2A lysine 119 (H2AK119) deubiquitinase in drosophila [9] and was then further characterized as a tumor suppressor in leukemogenesis [10]. BAP1 complex functions by binding to both promoters and enhancers at the chromatin level [4] and acts as a general transcriptional activator via its deubiquitinase activity on histone H2A monoubiquitination in drosophila [11]. In addition, BAP1 complex could mediate the chromatin recruitment of other epigenetic complexes, such as MLL3/COMPASS [4] to activate transcription. On the other hand, the enzymatic activity of BAP1 is also required for the malignant progression of some cancer types [12, 13], which express high levels of RNF2 [14].

The BAP1 complex is a multi-protein complex, containing as many as eleven different subunits. The additional sex combs-like proteins (ASXL1–3) were found as essential core subunits within BAP1 complex [15]. The primary function for ASXLs is to stabilize and link BAP1 complex to the nucleosome [16]. ASXL1–3 proteins form mutually exclusive complexes with BAP1, due to a sequence similarity present at the N-terminal domain of ASXLs, which directly interacts with BAP1’s C-terminal domain (CTD) [11, 16, 17]. In contrast to the ubiquitous expression of ASXL1 and ASXL2 across different tissue types, ASXL3 expression pattern is shown to be more tissue-specific [18]. However, the functions and chromatin localization of each ASXL protein, as well as how cells are able to control the stoichiometry between ASXL1/BAP1, ASXL2/BAP1, and ASXL3/BAP1, remain unknown.

Lung cancer is the leading cause of cancer deaths in men and the second leading cause of cancer deaths in women worldwide [19]. In the USA, it is estimated that for lung cancer there was a total of 228,150 new cases and 142,670 deaths in 2019 [20]. Lung cancer is classified as small cell lung carcinoma (SCLC) (around 13%) or non-small cell lung carcinoma (NSCLC) (around 83% of cases) [20]. SCLC is characterized as being more aggressive and a deadlier form of lung cancer with a predisposition for rapid growth, early metastasis, and acquired therapeutic resistance [21, 22]. Thus, SCLC is rarely localized at diagnosis, and as a result, tumor resection is no longer a viable treatment option. Instead, the only option for most patients with SCLC is to revert to chemotherapy; however, this can lead to poor prognosis due to developed resistance to chemotherapy [22, 23]. In this study, we reported that the ASXL3/BAP1 complex is a tissue-specific PR-DUB complex that is exclusively expressed in SCLC. Mechanistically, we identified a specific BRD4 binding motif (BBM) within ASXL3 protein, which allows a physical and direct interaction between BRD4 and ASXL3/BAP1 complex. Consequently, depletion of ASXL3 impairs the levels of active enhancer marker H3K27Ac and causes a dramatic loss of BRD4 occupancy at enhancer chromatin binding sites, which result in further inactivation of the enhancer nearby genes. Furthermore, pharmacologically induced inhibition of BET protein with BET chemical degrader (dBET6) [24] selectively inhibits highly expressed ASXL3 subtype SCLC cells by decreasing viability, indicating the dependency of the oncogenic function of ASXL3/BRD4 axis in human SCLC.

## Methods

### Antibodies and reagents

BAP1 (#13271S), CBX3 (#2619S), BRD4 (#13440S), CDK9 (#2316S), H3K27Ac (#8173S), H2AK119Ub (#8240), NSD3 (#92056S), JMJD6 (#60602S), CHD4 (#12011S), CCNT1 (#81464S), Pol II (#14958), histone H3 (#4499S), H3K4me1 (#5326S), and BRD2 (#5848S) antibodies were purchased from Cell Signaling. HSP90 (sc-7947) and GFP (sc-9996) antibodies were purchased from Santa Cruz. HCFC1 (A301-399A), BRD3 (A302-368A), and FOXK1 (A301-728A) antibodies were purchased from Bethyl Laboratories. Tubulin antibody (E7) was purchased from Developmental Studies Hybridoma Bank. Flag (F3165) antibody was purchased from Sigma. ASXL3 antibodies were made in-house with antigen peptides against ASXL3 (AA 1250-1399) and ASXL3 (AA 1405-1699). BAP1 ChIP-seq antibody was generated as described previously [4]. JQ1 (1268524-70-4) was purchased from MedChemExpress. Both dBET6 (S8762) and IBET-151 (S2780) were purchased from Selleckchem.

### Cell lines

HEK293T cells were obtained from ATCC and then maintained with DMEM (Gibco, Gaithersburg, MD) containing 10% FBS (Sigma). The SCLC cell lines were obtained from ATCC. NCI-H748, NCI-H1963, NCI-H209, NCI-H889, and NCI-H69 cells were maintained with ATCC-formulated RPMI-1640 medium containing 10% FBS (Sigma). NCI-H1882, NCI-H1436, NCI-H1105, and NCI-H2171 cells were maintained with ATCC-formulated DMEM/F12 cell culture media containing 10% FBS (Sigma).

### Immunoprecipitation (IP)

Cells were lysed in Triton lysis buffer (50 mM Tris pH 8.0, 150 mM NaCl, 0.5% Triton X100, 10% Glycerol, 1 mM DTT, protease inhibitors, and benzonase). After centrifugation at 20,000*g* for 15 min, the supernatants were collected and incubated with primary antibody at 4 °C for 2 h with rotation. After incubation with immobilized Protein A/G (Santa Cruz), samples were washed with lysis buffer four times, and proteins were resuspended in 5× SDS sample loading buffer and subjected to SDS-PAGE electrophoresis. The resolved proteins were either transferred to nitrocellulose membranes for immunoblotting or subjected to mass spectrometry analysis.

### RNA interference and real-time PCR

The cells were infected with lentivirus containing short-hairpin RNAs (shRNAs) in the presence of 4 μg/ml Polybrene (Sigma) for 24 h in DMEM supplemented with 10% FBS. The infected cells were selected with 2 μg/ml puromycin for an additional 48 h. The shRNA constructs were purchased from Sigma. The clone IDs for ASXL3 are TRCN0000246266 (sh*ASXL3*-#1) and TRCN0000246268 (sh*ASXL3*-#2). The non-targeting (sh*Ctrl*) shRNA construct (SHC002) was purchased from Sigma. Primers are listed in Additional file 1: Table S1.

### Plasmids

Three overlapped fragments of human ASXL1 were amplified from the human full-length ASXL1 DNA (MHS6278-213245938, GE Open Biosystems) and then inserted into pLNCX-GFP vector via Gibson assembly (NEB) at HpaI enzymatic restriction sites (GTT^AAC). The full-length ASXL2 was amplified from HEK293T cells and then inserted into pLNCX-GFP vector via Gibson assembly (NEB) at HpaI enzymatic restriction sites. The different ASXL3 truncations were amplified from pcDNA3-ASXL3-V5/His vector and then subcloned into pLNCX-GFP vector via Gibson assembly (NEB) at HpaI enzymatic restriction sites. The different BRD4 truncations were amplified from pcDNA5-Flag-BRD4 vector and then subcloned into pLNCX-Flag vector via Gibson assembly (NEB) at HpaI enzymatic restriction sites. Details of the cloning and primer sequences were listed in Additional file 1: Table S1.

### RNA-seq

Paramagnetic beads coupled with oligo d(T) are combined with total RNA to isolate poly(A)+ transcripts based on NEBNext® Poly(A) mRNA Magnetic Isolation Module manual. Prior to first strand synthesis, samples were randomly primed (5′ d(N6) 3′ [N=A,C,G,T]) and fragmented based on the manufacturer’s recommendations (NEBNext® Ultra™ II RNA Nondirectional Library Prep Kit for Illumina®). The first strand is synthesized with the Protoscript II Reverse Transcriptase with a longer extension period (40 min for 42 °C). All remaining steps for library construction were used according to the NEBNext® Ultra™ II RNA Nondirectional Library Prep Kit for Illumina®. Illumina 8-nt dual-indices were used. Samples were pooled and sequenced on a HiSeq with a read length configuration of 150 PE.

### RNA-seq analysis

Gene counts were computed by HTSeq [25] and used as an input for edgeR 3.0.852 [26]. Genes with Benjamini-Hochburg adjusted *p* values less than 0.01 were considered to be differentially expressed (unless otherwise specified). RNA-seq heatmaps adjacent to ChIP-seq heatmaps display log2 (fold change) values of genes corresponding to TSSs nearest to ChIP-seq peaks and were displayed using Java TreeView [27]. GO functional analysis was carried out using Gene Set Enrichment Analysis [28] and Metascape with default parameters [29]. The read counts of RNA-seq data from SCLC cell lines were downloaded from https://portals.broadinstitute.org/ccle/data [30] and analyzed using DESeq2 [31].

### ChIP-seq assay

Crosslinking: Cells were harvested and washed twice with ice-cold PBS and then fixed with paraformaldehyde (1% final) for 10 min at RT. Afterwards, the paraformaldehyde solution was quenched with 2.5 M (1/20) glycine, and then, cell pellets were washed twice with PBS. Sonication: The cell pellets were resuspended with lysis buffer 1 (50 mM HEPES, pH = 7.5, 140 mM NaCl, 1 mM EDTA, 10% Glycerol, 0.5% NP-40, 0.25% Triton X-100, 1X protease inhibitors) and then incubated on nutator at 4 °C for 10 min. Afterwards, cell pellets were centrifuged at 500 g for 5 min and discarded supernatant. Then, cell pellets were washed with lysis buffer 2 (10 mM Tris-HCl, pH = 8.0, 200 mM NaCl, 1 mM EDTA, 0.5 mM EGTA, 1 X protease inhibitors) and resuspended with lysis buffer 3 (10 mM Tris-HCl, pH = 8.0, 1 mM EDTA, 0.1% SDS, 1 X protease inhibitors). The final volume was adjusted to be 10 times the size of each cell pellet with lysis buffer 3. Sonication was performed with 1-ml Covaris tubes which were set to 10% duty factor, 175 peak intensity power, and 200 cycles per burst for 60–1200 s. Ten percent of 10X ChIP dilution buffer (10% Triton x-100, 1 M NaCl, 1% Na-Deoxycholate, 5% N-Lauroylsarcosine, 5 mM EGTA) was added to the lysate, and samples were centrifuged at maximum speed for 15 min at 4 °C to pellet debris. Immunoprecipitation: Antibody was added (~ 10 μg per purified antibody or 40 μl of anti-sera) to each sample. After incubation at 4 °C on nutator overnight, 100 μl Protein A/G Agarose beads were added for each sample for 2 h. The agarose beads were washed 4 times with RIPA buffer (50 mM HEPES, pH = 7.5, 500 mM LiCl, 1 mM EDTA, 1.0% NP-40, 0.7% Na-Deoxycholate), followed by once with ice-cold TE buffer (with 50 mM NaCl). After removing the residual buffer, the DNA for each IP sample was eluted with elution buffer (50 mM Tris-HCl, pH = 8.0, 10 mM EDTA, 1.0% SDS) and reverse cross-linked at 65 °C oven for 6–15 h, followed by protease K digestion at 55 °C for 2 h. The genomic DNA fragments were then further purified with Qiagen DNA purification kit (Cat. No. 28104).

### ChIP-seq analysis

For ChIP-seq analysis, all the peaks were called with the MACS v1.4.2 software [32] using default parameters and corresponding input samples. Metaplots and heatmaps were generated using ngsplot database [33] to display ChIPseq signals aligned with ASXL3-specific peaks, which is defined by overlapping peaks found within both antibodies against ASXL3 using BEDTools [34]. Peak annotation, motif analysis, and super enhancer analysis were performed with HOMER [35]. Correlation of ASXL3 ChIP-seq was analyzed with deepTools [36]. Both TSS and non-TSS were clustered based on the peak annotation from HOMER.

### Mass spectrometry sample preparation

Protein pellet was denatured in 50 μL of 8 M Urea/0.4 M Ammonium Bicarbonate followed by reduction in 2 μL of 100 mM DTT. Protein was alkylated with 18 mM iodoacetamide for 30 min at room temperature in the dark. Samples were diluted with four volumes of water to bring urea concentration to 1.8 M. Sequencing-grade trypsin (Promega) was added at 1:100 (enzyme: substrate) and incubated at 37 °C overnight. The digests were acidified to 0.5% trifluoroacetic acid (TFA), and the peptides were desalted on C18 Sep-Paks (Waters). Peptides were eluted with 2X 50 μL of 80% ACN/0.1% TFA to ensure complete recovery. The pooled extracts were dried in a vacuum concentrator and resuspended in 30 μL of 5% ACN/0.1% FA for LC-MS analysis.

### LC-MS/MS analysis

Peptides were analyzed by LC-MS/MS using a Dionex UltiMate 3000 Rapid Separation LC (RSLC) systems and a linear ion trap—Orbitrap hybrid Elite mass spectrometer (Thermo Fisher Scientific Inc., San Jose, CA). Six-microliter peptide samples were loaded onto the trap column, which was 150 μm × 3 cm in-house packed with 3 μm ReproSil-Pur® beads (New Objective, Inc. Woburn, MA). The analytical column was a 75 μm × 10.5 cm PicoChip column packed with 3-μm ReproSil-Pur® beads. The flow rate was kept at 300 nL/min. Solvent A was 0.1% FA in water, and Solvent B was 0.1% FA in ACN. The peptide was separated on a 120-min analytical gradient from 5% ACN/0.1% FA to 40% ACN/0.1% FA. The mass spectrometer was operated in data-dependent mode. The source voltage was 2.40 kV, and the capillary temperature was 275 °C. MS1 scans were acquired from 400 to 2000 m/z at 60,000 resolving power and automatic gain control (AGC) set to 1 × 106. The top fifteen most abundant precursor ions in each MS1 scan were selected for fragmentation. Precursors were selected with an isolation width of 1 Da and fragmented by collision-induced dissociation (CID) at 35% normalized collision energy in the ion trap; previously selected ions were dynamically excluded from re-selection for 60 s. The MS2 AGC was set to 3 × 105.

### Mass spectrometry data analysis

Proteins were identified from the MS raw files using Mascot search engine (Matrix Science, London, UK. version 2.5.1). MS/MS spectra were searched against the SwissProt human database. All searches included carbamidomethyl cysteine as a fixed modification and oxidized Met, deamidated Asn and Gln, and acetylated N-term as variable modifications. Three missed tryptic cleavages were allowed. The MS1 precursor mass tolerance was set to 10 ppm, and the MS2 tolerance was set to 0.6 Da. A 1% false discovery rate cutoff was applied at the peptide level. Only proteins with a minimum of two peptides above the cutoff were considered for further study.

### Statistical analyses

For statistical analyses, GraphPad Prism 7, Microsoft Excel, and R were used. All data involving a statistical analysis being reported that met the criteria to use the appropriate statistical tests; for the normal distribution of data, the empirical rule was used to infer the distribution. For growth curves and time-course, RNA-seq *t* tests were calculated between the area-under-the-curve (AUC) values. Statistical tests used are reported in the figure legends.

## Results

### The Polycomb group (PcG) protein ASXL3 defines a subtype of small cell lung cancer (SCLC) with high expression of ASCL1

**Fig. 1:**
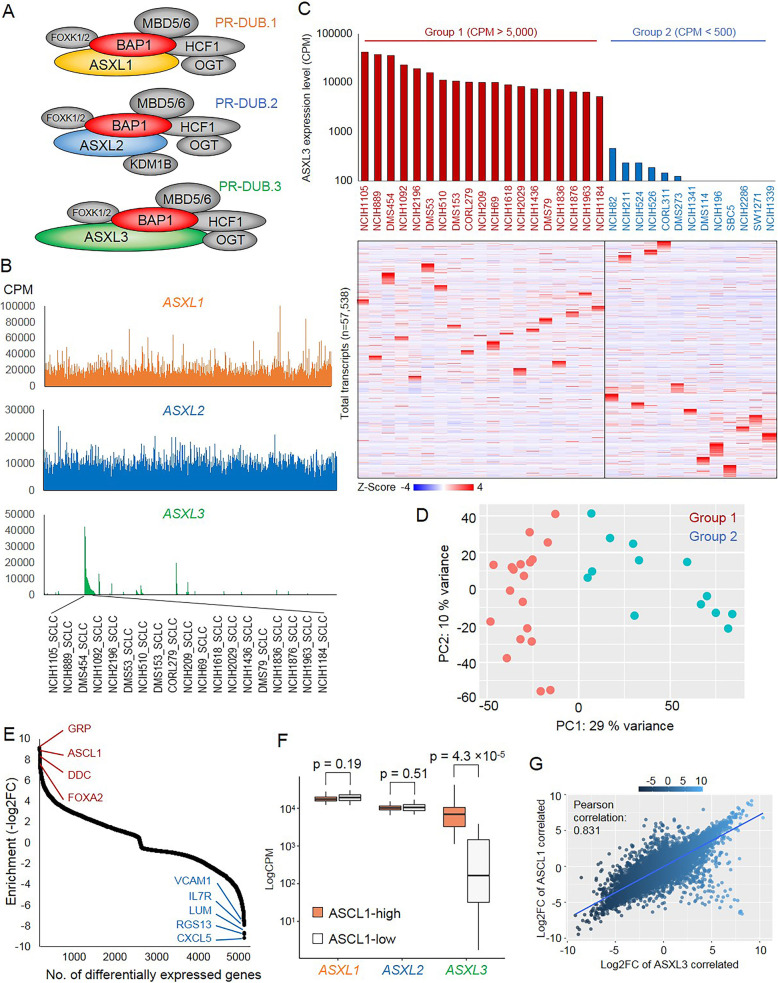
ASXL3 defines a subtype of SCLC with high expression of ASCL1. **a** Schematic of three BAP1 complexes, which is defined by 3 different additional sex-combs-like proteins ASXL1, ASXL2, and ASXL3. **b** The counts per million (CPM) value for ASXL1/2/3 expression in 1004 human cell lines. **c** The log2 (fold-change) heatmaps show the global gene expression profile in ASXL3-high (CPM > 5000) and ASXL3-low (CPM < 500) SCLC cell lines. **d** The PCA plot analysis indicated that the ASXL3-high and ASXL3-low cell lines are separated into two distinctive groups of SCLC cell types. **e** The gene expression enrichment analysis shows the most enriched genes in ASXL3-high and ASXL3-low SCLC cell lines. **f** The box plot shows the expression level of ASXL1, ASXL2, and ASXL3 between ASCL1-high and ASCL1-low SCLC cell lines. *P* value is calculated based on the *t* test. **g** The scatter plot shows the correlation between ASXL3-associated genes and ASCL1-associated genes via log2 fold-change

The additional sex-combs-like proteins ASXL1, ASXL2, and ASXL3 determine the activity and stability of BAP1 complex (Fig. 1a). However, the expression patterns of all three *ASXLs* across different tumor types were poorly understood. To determine relative expression of the three different ASXL proteins, we retrieved the published RNA-seq data from 1004 human cell lines [30] and then further compared *ASXLs*’ expression between different cell lines. As a result, we found *ASXL1* and *ASXL2* were widely expressed in a vast majority of cell lines (Fig. 1b). However, *ASXL3* was only expressed in a few cell lines, especially in a cluster of SCLC cell lines (Fig. 1b). Interestingly, a recent study has reported that *ASXL3* overexpression is correlated with an increased genomic copy number in SCLC cell lines and is essential for SCLC cell viability [18]. These results revealed that ASXL3 is highly tissue-specific, which may have a distinct function, in comparison to both ASXL1 and ASXL2. To further understand the role of ASXL3 in SCLC, we retrieved the RNA-seq data from 50 SCLC cell lines and then divided the cell lines into two different groups, based on *ASXL3* expression (*ASXL3*-high, CPM of *ASXL3* > 5000 and *ASXL3*-low, CPM of *ASXL3* < 500). As shown in Fig. 1c, we found that the global transcriptome (*n* = 57,538) of these two groups of SCLC cell lines were distinct from one another, which was also supported by the principal component analysis (PCA), since the first principal component was sufficient to separate group 1 and group 2 SCLC cell lines (Fig. 1d). Overall, we found that 2632 genes were significantly enriched in the *ASXL3*-highly expressed group of SCLC cells and 2721 genes were significantly enriched in the *ASXL3*-lowly expressed group of SCLC cells (adj. *p* < 0.05) (Additional file 2: Figure S1A, Additional file 3: Table S2). In contrast, there was no obvious change in expression for *ASXL1/2* that correlated with *ASXL3*-high and *ASXL3*-low SCLC cell lines (Additional file 2: Figure S1B). Among all of the genes that were positively correlated with ASXL3 expression, we found that the expression of achaete-scute family bHLH transcription factor 1 (ASCL1), a lineage oncogenic transcription factor in SCLC [37], was enriched approximately 400 times as much in *ASXL3*-high group SCLC cell lines compared to *ASXL3*-low group SCLC cell lines (Fig. 1e and Additional file 2: Figure S1C) and vice versa (Additional file 2: Figure S1D). Interestingly, there is no significant difference in the *ASXL1/2* expression between *ASCL1*-high and *ASCL1*-low SCLC cells in contrast to *ASXL3* expression (Fig. 1f). Thus, *ASXL3*-high SCLC cells might have a similar feature as *ASCL1*-high SCLC cells, such as being essential for neuroendocrine (NE) lung cancer development. Notably, the gene expression patterns between *ASXL3*-high and *ASCL1*-high SCLC cells were shown to be significantly correlated (*R* = 0.831) (Fig. 1g). Pathway analysis indicated that multiple neuron differentiation signaling is significantly enriched within 1484 genes (Additional file 2: Figure S1E and S1F), which were positively correlated with both *ASXL3* and *ASCL1* in SCLC. However, knockdown of *ASCL1* did not affect *ASXL3* expression levels (Additional file 2: Figure S1G). This result suggested that ASXL3 is an independent biomarker for SCLC-A type, which is defined by *ASCL1* expression [38]. Notably, as classical neuroendocrine (NE) markers, the expression of both decarboxylase (DDC) and gastrin-releasing peptide (GRP) were also significantly enriched in *ASXL3*-highly expressed SCLC cells [39] (Fig. 1e).

### BRD4 directly interacts with ASXL3 in small cell lung cancer cells (SCLC)

**Fig. 2:**
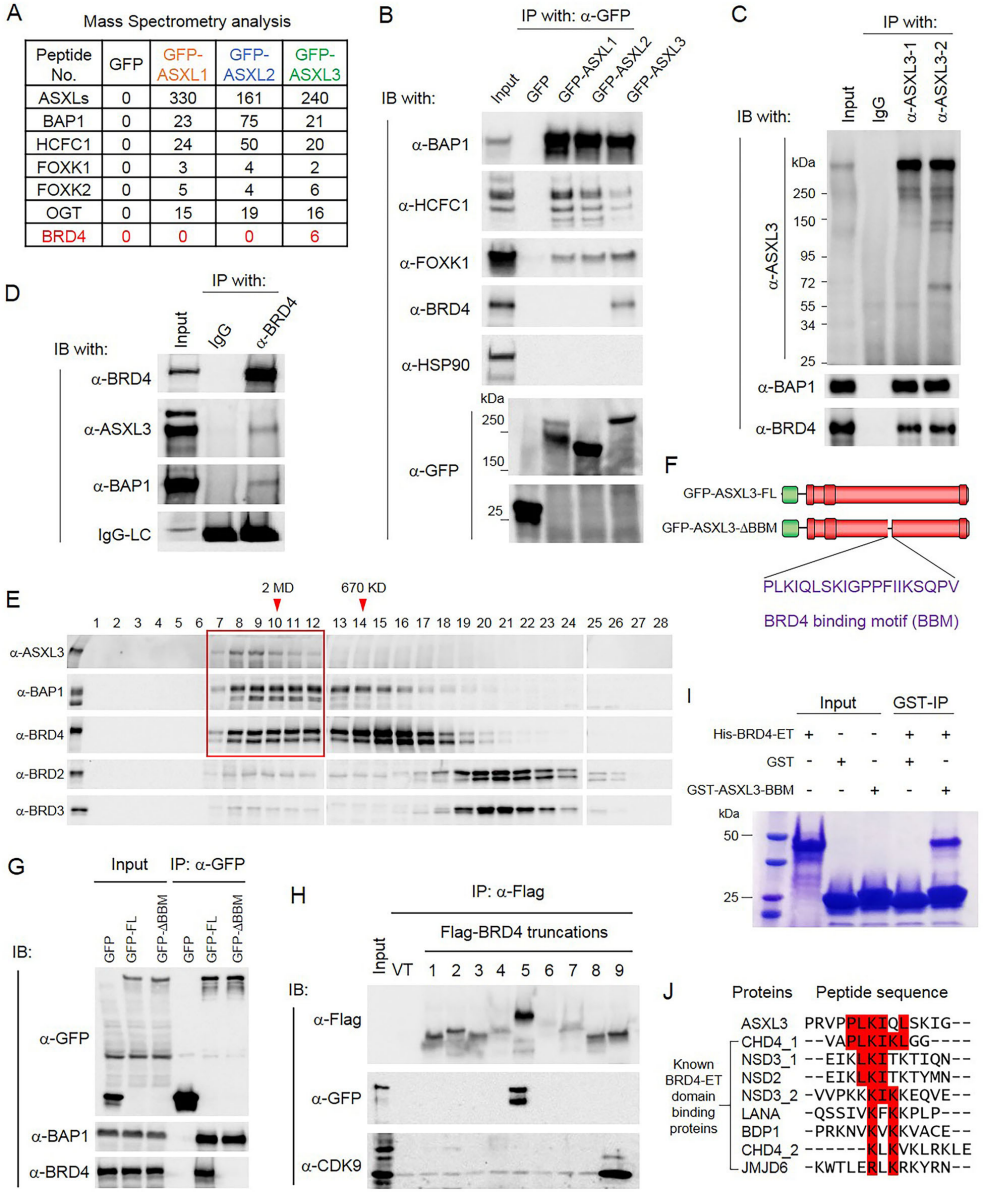
ASXL3 directly interacts with BRD4 in small cell lung cancer cells. **a** The GFP-fusion proteins were purified from HEK293T cells transfected with GFP-tagged ASXL1/2/3. The purified proteins were subjected to mass spectrometry analysis. Peptide numbers of BAP1 complex subunits and BRD4 pulled down by GFP-ASXLs were shown. **b** BAP1, HCF1, FOXK1, and BRD4 levels in HEK293T cells 24 h after transfection with plasmids expressing GFP or GFP-ASXL1–3. Immunoprecipitation (IP) from whole cell lysates were performed with antibodies against the GFP epitope, followed by immunoblotting (IB) with antibodies against the indicated proteins. HSP90 was used as negative control, *n* = 3. **c** IP of endogenous ASXL3 of NCI-H1963 cells with two different homemade antibodies followed by IB for BAP1 and BRD4; IgG was used as negative control, *n* = 3. **d** IP of endogenous BRD4 from NCI-H1963 cells followed by IB for BRD4, ASXL3, and BAP1; IgG was used as negative control, *n* = 3. LC, light chain. **e** Nuclear extract from NCI-H1963 SCLC cells was subjected to size exclusion chromatography, and then protein levels of ASXL3, BAP1, and BRD4 were determined by western blot analysis. BRD2 and BRD3 were used as controls, *n* = 2. **f** Schematic diagram depicting the BBM depletion of human ASXL3 protein. **g** BAP1 and BRD4 levels in HEK293T cells 24 h after transfection with plasmids expressing GFP, GFP-ASXL3-FL, or GFP-ASXL3-ΔBBM. IP from whole cell lysates was performed with antibodies against the GFP epitope, followed by IB with antibodies against the BAP1 and BRD4, *n* = 3. **h** 293T cells were transfected with Flag-tagged BRD4 truncations (Additional file 2: Figure S3B) together with GFP-tagged ASXL3. Whole-cell lysates were used for IP with Flag antibody followed by IB for GFP. CDK9 was used as positive control, *n* = 3. **i** Recombinant His-tagged BRD4-ET domain and GST-tagged ASXL3-BBM was purified from *Escherichia coli*. The in vitro binding assay was performed to determine the direct interaction between BRD4-ET domain and ASXL3-BBM, *n* = 3. **j** Alignment by CLUSTALW analysis shows the similarity between ASXL3-BBM and BRD4 binding motif within other BRD4 binding proteins

The three mammalian homologs of the Drosophila Asx, ASXL1–3, all share a similar N-terminal (ASXN), ASXM, and C-terminal (PHD) domains (Additional file 2: Figure S2A). To determine the interactome of ASXL1, ASXL2, and ASXL3, we purified GFP-tagged ASXLs from HEK293T cells (Additional file 2: Figure S2B). By mass spectrometry analysis, we identified that the full-length ASXL3 specifically interacted with bromodomain-containing protein 4 (BRD4), which binds directly to acetylated histones at enhancers and promoters via its bromodomains, to regulate transcriptional elongation [40] (Fig. 2a, Additional file 4: Table S3). Interestingly, there was no detectable interaction between ASXL1/2 and BRD4, indicating that ASXL3 exclusively interacts with BRD4 (Fig. 2a). To validate our mass spectrometry result, we performed co-immunoprecipitation against GFP-tagged ASXLs and found all of the three different ASXL proteins were equally bound to BAP1 and other subunits within BAP1 complex, such as FOXK1 and HCF1 [41] (Fig. 2b). Consistent with the mass spectrometry result, we found ASXL3 (but not ASXL1/2) interacted with endogenous BRD4 (Fig. 2b). To determine the endogenous protein-protein interaction between ASXL3 and BRD4, we generated two different polyclonal antibodies against different regions of ASXL3 (Additional file 2: Figure S2C). To validate the specificity of both antibodies, we first performed a peptide competition assay to determine the specificity of both homemade polyclonal antibodies (Additional file 2: Figure S2D). Then, we knocked down ASXL3 with two distinct shRNAs in human SCLC cell line NCI-H1963, which expresses high levels of ASXL3 protein (Additional file 2: Figure S2E). Based on western blot analysis, both of our antibodies specifically recognize endogenous ASXL3 protein (Additional file 2: Figure S2F). In addition, we observed that loss of ASXL3 in SCLC cells significantly reduced cell growth and colony formation ability in vitro (Additional file 2: Figure S2G). Using these two homemade anti-ASXL3 antibodies, we were able to immunoprecipitate endogenous ASXL3 and detect BAP1 and BRD4 as co-bounded proteins (Fig. 2c). We further performed a reciprocal immunoprecipitation using an anti-BRD4 antibody and found that both BAP1 and ASXL3 could be detected in the immunoprecipitates (Fig. 2d). The endogenous protein-protein interaction between ASXL3 and BRD4 was also confirmed in mouse SCLC cell line KP3 cells, which has a phenotype that lacks functional p53 and RB [42, 43] (Additional file 2: Figure S2H). In HEK293T cells, which express very low levels of ASXL3, transfection with GFP-tagged ASXL3 showed a dose-dependent increase in the protein-protein interaction between BAP1 and BRD4 (Additional file 2: Figure S2I). Finally, to study the stoichiometry of endogenous ASXL3 and BRD4 interaction, nuclear extracts from NCI-H1963 cells were subject to size exclusion chromatography, followed by western blot analysis of the elution profile of ASXL3, BAP1, and the bromodomain proteins BRD2, BRD3, and BRD4 (Fig. 2e). These studies clearly indicated that a significant proportion of BRD4, but not BRD2 or BRD3, co-eluted with ASXL3/BAP1 complex at approximately 2 M Da within ASXL3-highly expressed SCLC cells (Fig. 2e).

Based on the above findings, we sought to determine whether there is a direct interaction between ASXL3 and BRD4. This was accomplished by creating a series of eighteen constructs that tile across full-length ASXL3 (Additional file 2: Figure S3A). The GFP-tagged series were expressed in 293T cells (Additional file 2: Figure S3B), and fragments F2, F7, F9, F10, F11, F15, F16, and F17 could pull-down flag-tagged BRD4 (Additional file 2: Figure S3C). Consistent with the IP experiment, a mass spectrometry data of BRD4 interacting with fragments of ASXL3 also supported the conclusion (Additional file 2: Figure S3D, Additional file 4: Table S3). Ultimately, we narrowed down to a region containing 20 amino acids of ASXL3 that is essential for ASXL3/BRD4 binding and thus named it BRD4 binding motif (BBM) (Fig. 2f). Furthermore, depletion of the 20 amino acids (ΔBBM) does not affect ASXL3/BAP1 interaction, but only completely abolished ASXL3/BRD4 interaction in HEK293T cells (Fig. 2g). Interestingly, ASXL3 homolog was not found in zebrafish, but instead ASXL3 BBM is found to be highly conserved from frogs to mammals (Additional file 2: Figure S3E). BRD4 is a transcriptional and epigenetic regulator that plays a pivotal role during transcription. The bromodomains at the N-terminus of BRD4 binds to acetylated histones and transcription factors. The C-terminus of BRD4 is involved in the chromatin recruitment of positive transcription elongation factor b (p-TEFb) complex, which is essential for transcription elongation. To determine which functional domain within BRD4 interacts with ASXL3, we truncated BRD4 into several fragments based on a previous study [44] (Additional file 2: Figure S3F). As shown in the co-immunoprecipitation experiment, we found that the full-length ASXL3 binds to the extra-terminal (ET) domain of BRD4 (Fig. 2h). The ET domain of BRD4 is well known to mediate the interaction between BRD4 and numerous epigenetic factors such as JMJD6 [45], NSD3 [46], and CHD4 [47]. Therefore, ASXL3 is a novel and tissue-specific BRD4-ET domain binding protein. To demonstrate whether there is a direct binding between ASXL3-BBM and BRD4-ET domain, we performed an in vitro binding assay with recombinant his-tagged BRD4-ET domain and GST-tagged ASXL3-BBM and further showed the direct interactions between both recombinant proteins (Fig. 2i). Then, we further compared the ASXL3-BBM domain among other known BRD4 binding proteins [48] and found that the amino acid sequences from these BRD4 binding proteins share similarities with ASXL3-BBM (Fig. 2j).

### Genomic co-localization of ASXL3 and BRD4 in SCLC

**Fig. 3:**
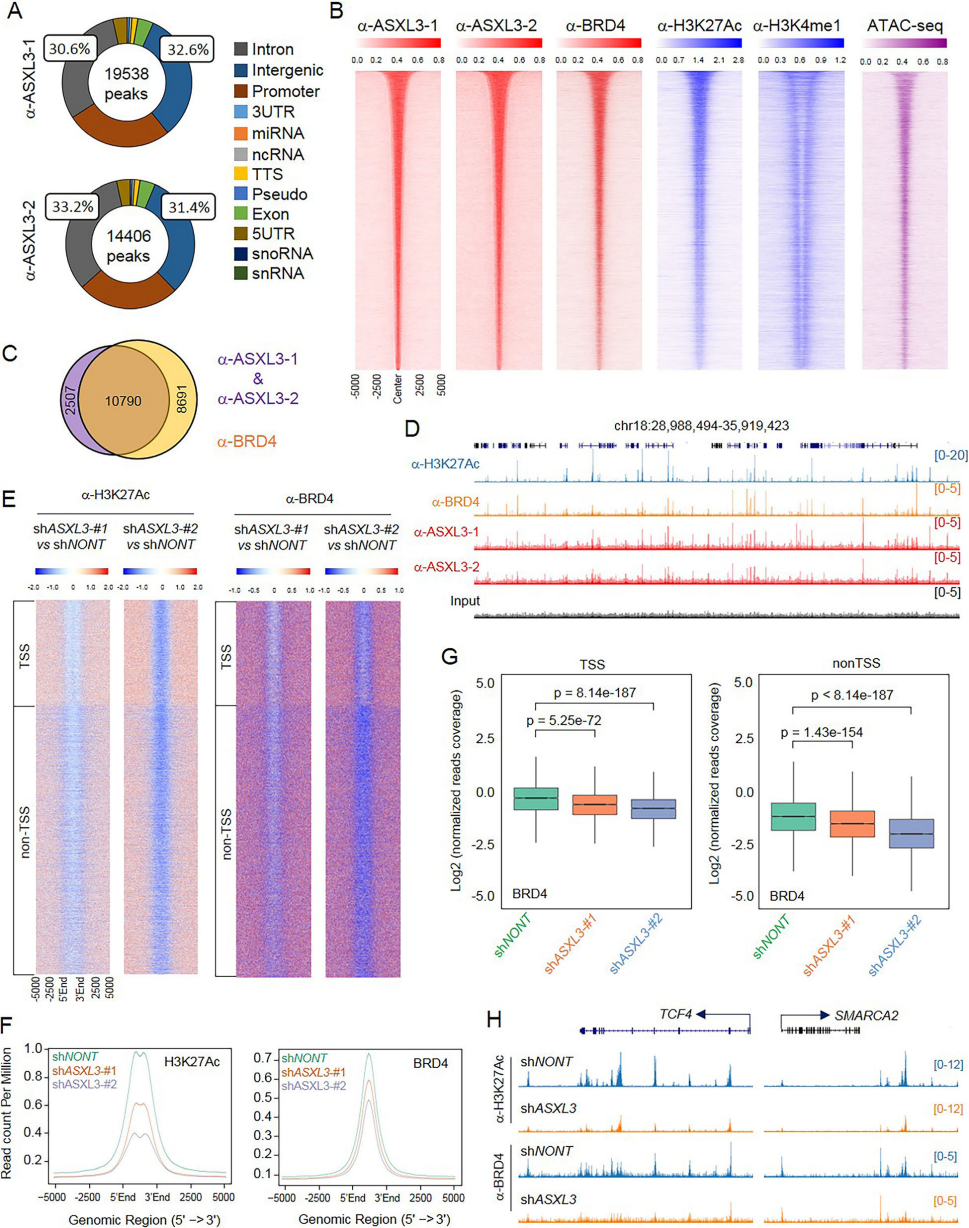
Genomic co-localization of ASXL3 and BRD4 in SCLC. **a** Distribution of ASXL3 binding to genomic regions in the human SCLC cell line NCI-H1963, as assessed by chromatin immunoprecipitation sequencing (ChIP-seq) using two different ASXL3 specific antibodies. Peak annotation of ASXL3 is summarized in a pie chart format. **b** Sorted and centered heatmaps generated from ChIP-seq data analyses show the occupancy of ASXL3, BRD4, H3K27Ac, and H3K4me1 in NCI-H1963 SCLC cells. All rows are centered on ASXL3 peaks based on the ranking of signals. The published ATAC-seq data from the same cell line (GSM3321013) was also centered on ASXL3 peaks. **c** A Venn-diagram presentation of the overlap between ASXL3 and BRD4 peaks. **d** Representative tracks showing chromatin occupancy of H3K27Ac, BRD4, and ASXL3 binding sites. **e** The log2 fold-change heatmaps show the occupancy of H3K27Ac levels (left panel) and BRD4 levels (right panel) in cells transduced with ASXL3 shRNAs or non-targeting shRNA at TSS and non-TSS regions. **f** The average plot shows the global reduction of H3K27Ac (left panel) and BRD4 (right panel) in ASXL3-depleted cells. **g** The box plot shows the BRD4 occupancy in cells transduced with ASXL3 shRNAs or non-targeting shRNA at TSS and non-TSS regions. **h** Representative tracks showing chromatin occupancy of H3K27Ac and BRD4 at TCF4 (left) or SMARCA2 (right) gene loci

To determine the function of ASXL3 as an epigenetic factor in SCLC cells genome-wide, we performed ChIP-seq with two different homemade antibodies against ASXL3. The pie-plots shown in Fig. 3a revealed that 19,538 and 13,406 ASXL3 peaks were detected by each antibody, respectively. The average plot analysis revealed the chromatin binding patterns of the peaks detected by both antibodies are similar (Additional file 2: Figure S4A and S4B). Genome-wide annotation analysis shows that more than 60% of ASXL3 peaks localizes at introns and intergenic regions (Fig. 3a). Motif enrichment analysis of ASXL3 peaks demonstrated an enrichment of Six-1 and B-MYB transcription factor (TF) binding motifs—both of which have been found to be critical for lung tumorigenesis (Additional file 2: Figure S4C). To further elucidate ASXL3 functions at enhancers, we performed BRD4, H3K27Ac, and H3K4me1 ChIP-seq in NCI-H1963 SCLC cells. As a result, we found a significant amount of co-localization between ASXL3 and BRD4 at active enhancer regions, where high levels of H3K27Ac and H3K4me1 were significantly enriched (Fig. 3b). Intriguingly, there were 10,790 peaks overlapped between ASXL3 and BRD4 (Fig. 3c and d). We further retrieved the ATAC-seq data, which enables the study of chromatin openness and interplay between TFs and accessible chromatin regions, from NCI-H1963 SCLC cell line (GSM3321014) [49]. Genome-wide ATAC-seq analysis revealed that ASXL3 and BRD4 co-localized at open chromatin loci (Fig. 3b and Additional file 2: Figure S4D). To further determine the co-function between ASXL3 and BRD4 at the genome-wide level, we divided all ASXL3 peaks into transcriptional start site (TSS) and non-TSS regions and further centered BRD4 peaks on ASXL3 peaks at TSS and non-TSS loci. We found BRD4 was significantly enriched in both ASXL3’s TSS peaks and non-TSS peaks (Additional file 2: Figure S4E). GO pathway analysis demonstrated that ASXL3 TSS and non-TSS nearest genes are involved in metabolic and neural precursor cell proliferation pathways (Additional file 2: Figure S4F and S4G). Then, we sought to confirm our findings in mouse SCLC cell lines KP1 and KP3—which are p53 and RB mutant cells. By western blot analysis, we found both cell lines expressed high levels of ASXL3 protein (Additional file 2: Figure S4H). ChIP-seq analysis in KP3 cells showed a remarkable enrichment of H3K27Ac levels at ASXL3 occupied loci (Additional file 2: Figure S4I and S4J), which is consistent with our observations seen in human SCLC cells (Fig. 3b). Based on previous studies, most of the BRD4-ET domain binding proteins are involved in the functional activity or chromatin recruitment of BRD4. The co-localization of ASXL3 and BRD4 on chromatin suggests that BRD4 recruitment may be determined by interactions with ASXL3. To test this possibility, we performed ChIP-seq in ASXL3-WT and ASXL3-depleted cells with BRD4 and H3K27Ac antibodies. Chromatin occupancies of BRD4 and H3K27Ac were centered on ASXL3 peaks at TSS and non-TSS regions. Loss of ASXL3 resulted in a remarkable reduction of H3K27Ac and BRD4 levels specifically at non-TSS regions (Fig. 3e), which exhibited an active enhancer signature of relatively high ratios for both H3K4me1 and H3K27Ac epigenetic markers (Fig. 3b). Genome-wide analysis was also performed to determine the total BRD4 and H3K27Ac levels at chromatin. We found loss of ASXL3 globally reduced H3K27Ac levels at chromatin, which may be due to the reduction of BRD4 chromatin recruitment (Fig. 3f). Comparing with TSS regions, we found a greater loss in BRD4 recruitment at non-TSS regions (Fig. 3g and h). In addition, we did not notice any decrease of total BRD4 protein levels in ASXL3-depleted cells, which further supports the notion that loss of ASXL3 does not affect BRD4 expression but instead may impact the epigenetic dynamics for BRD4 chromatin recruitment (Additional file 2: Figure S4K). Overall, these results demonstrated that ASXL3 marks active enhancers and mediates BRD4 recruitment to chromatin, suggesting that ASXL3 may play essential roles in transcription regulation at enhancers in both human and mouse SCLCs.

### Enhancer ASXL3 determines the expression of nearby genes

**Fig. 4:**
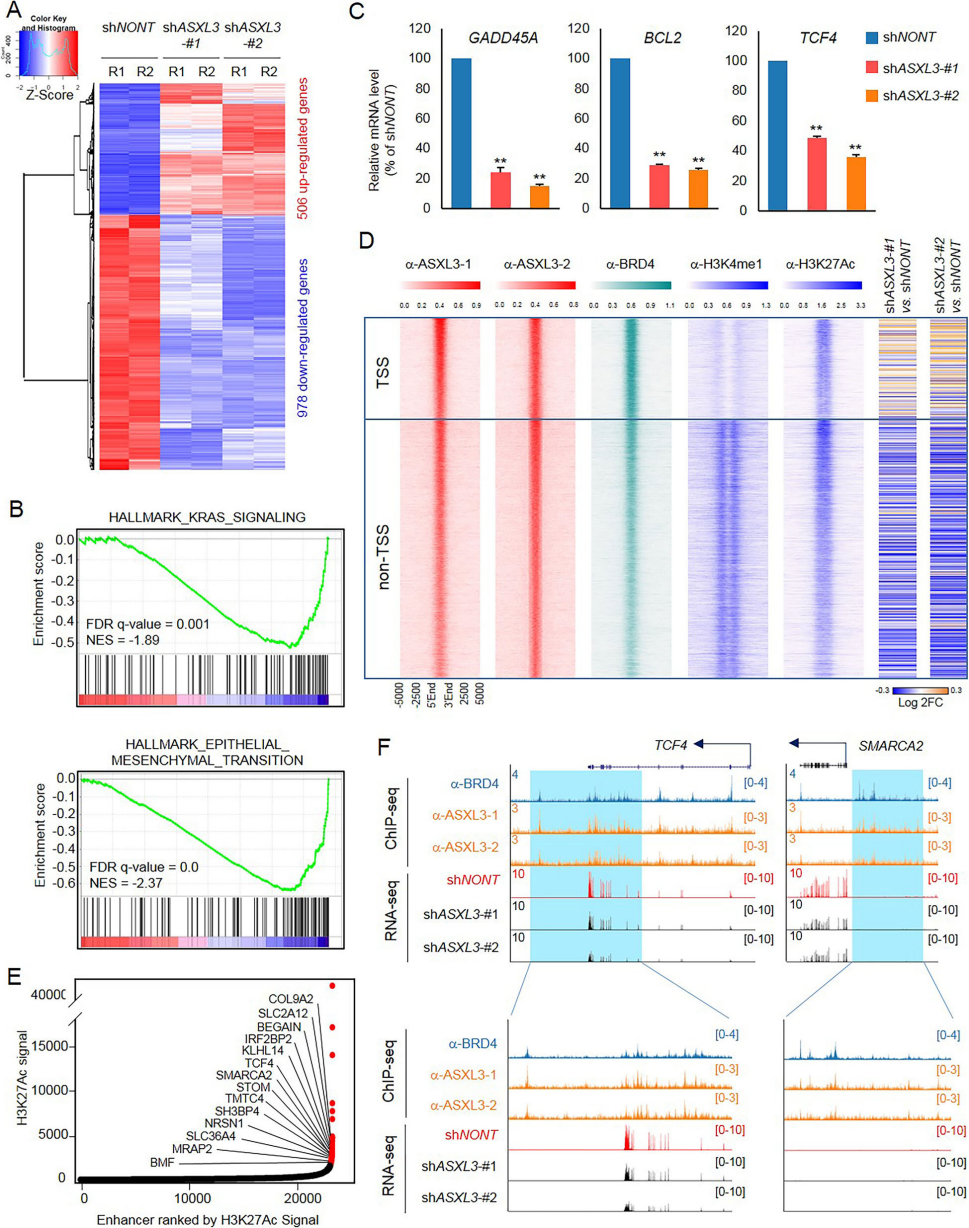
Enhancer ASXL3 determines the expression of nearby genes. **a** ASXL3 was knocked down by two different shRNAs. RNA-seq was performed for NCI-H1963 cells transduced with non-targeting shRNA, and two different ASXL3-specific shRNAs, *n* = 2. **b** GSEA analysis shows the most enriched gene expression signature in ASXL3-depleted cells. **c** Real-time PCR was performed to determine the relative gene expression of *GADD45A*, *BCL2*, and *TCF4* in NCI-H1963 cells transduced with non-targeting shRNA, and two different ASXL3-specific shRNAs, *n* = 3, two-tailed unpaired Student’s *t* test. **P < 0.01; *P < 0.05. **d** Heatmaps generated from ChIP-seq data analysis showing the occupancy of ASXL3, BRD4, H3K4me1, and H3K27Ac at ASXL3 binding sites. All rows are centered at ASXL3 peaks and then further divided into TSS and non-TSS regions. The right panel shows the log2 fold change of nearby gene expression in NCI-H1963 cells transduced with non-targeting shRNA or ASXL3-specific shRNAs, *n* = 2. **e** Histone H3 lysine 27 acetylation (H3K27ac) signals from chromatin immunoprecipitation (ChIP) sequencing identifies putative super enhancers (SEs) in NCI-H1963 cells. Hockey-stick plot representing the normalized rank and signals of H3K27Ac. Representative SE-associated genes that are controlled by ASXL3 are labeled. **f** Representative tracks showing the enhancer binding of ASXL3 and BRD4, which contributes to activation of gene expression

To investigate if ASXL3 loss at enhancers alters expression of nearby genes, we performed RNA-seq in SCLCs transduced with either non-targeting shRNA (sh*NONT*) or two ASXL3 distinct shRNAs to minimize off-target effects. A total of 978 genes were consistently downregulated, and 506 genes were upregulated in ASXL3-depleted cells (Fig. 4a). Gene Set Enrichment Analysis (GSEA) and Metascape pathway analysis showed multiple cell growth and differentiation signaling pathways that were significantly altered after ASXL3 depletion (Fig. 4b and Additional file 2: Figure S5A). Interestingly, we noticed the expression levels of a handful of canonical BRD4 target genes, such as *GADD45A*, *BCL2*, and *TCF4* being significantly reduced in ASXL3-depleted cells (Fig. 4c). We further knocked down ASXL3 within two other SCLC cell lines, NCI-H748 and NCI-H1882, with ASXL3-specific shRNAs (Additional file 2: Figure S5B) and confirmed the expression change of the indicated genes by real-time PCR (Additional file 2: Figure S5C). To determine the impact of ASXL3 on gene expression at enhancers, we integrated our RNA-seq data with ASXL3 ChIP-seq analysis separated by TSS and non-TSS clusters and examined the fold-change of genes nearest to ASXL3 peaks (Fig. 4d). As a result, loss of ASXL3 at active enhancer regions (enrichment of both H3K4me1 and H3K27Ac) was significantly correlated with reduction of nearest gene expression (Fig. 4d). In general, ASXL3 occupied the non-TSS region of 328 genes and TSS region of 116 genes, expression levels of which were downregulated in ASXL3-depleted cells (Additional file 2: Figure S5D, Additional file 5: Table S4). Based on pathway analysis, it is suggested that the function of ASXL3 at TSS and non-TSS regions might differ (Additional file 2: Figure S5E). In addition, a number of super enhancer (SE)-associated transcripts were identified in NCI-H1963 SCLC cells, which is based on the ranking of H3K27Ac ChIP-seq signals (Fig. 4e) or BRD4 ChIP-seq signals (Additional file 2: Figure S5F), and a number of ASXL3 downstream targets were associated with SEs, such as *TCF4*, *SMARCA2*, *COL9A2*, and *KLHL14* (Fig. 4e and f). In addition, there are 17 super enhancers that are co-occupied by both ASXL3 and BRD4 (Additional file 6: Table S5). Overall, our result directly demonstrated that ASXL3 is an enhancer binding factor, which is responsible for enhancer activity and the expression of nearby genes.

### ASXL3 is a direct target of BET inhibitors and predicts drug sensitivity

**Fig. 5:**
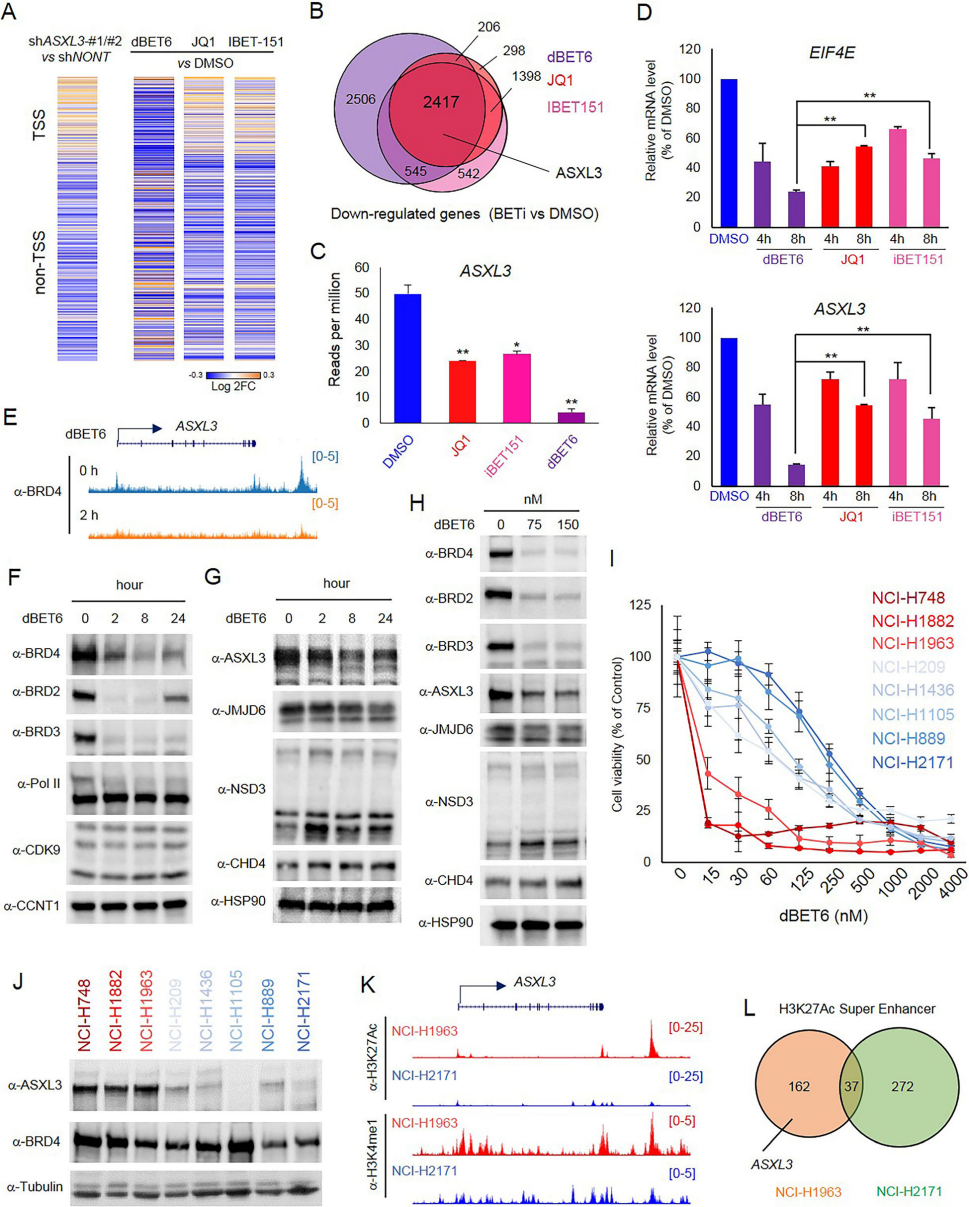
ASXL3 is a direct target of BET inhibitors and predicts drug sensitivity. **a** The log2 fold change of nearby gene expression in ASXL3-depleted cells, and cells treated with either DMSO, dBET6 (300 nM), JQ1 (1 μM), or IBET-151 (1 μM). **b** The Venn-diagram shows the overlap of downregulated genes in cells treated with dBET6, JQ1, or IBET-151 from (**a**). **c** The bar plot shows ASXL3 expression (RPM) in NCI-H1963 cells treated with different BET inhibitors, *n* = 2, two-tailed unpaired Student’s *t* test. **P < 0.01; *P < 0.05. **d** Real-time PCR was performed to determine the relative gene expression of EIF4E and ASXL3 in NCI-H1963 cells treated with dBET6, JQ1, or IBET-151 for 4 h or 8 h. DMSO was used as negative control, *n* = 3, two-tailed unpaired Student’s *t* test. **P < 0.01; *P < 0.05. **e** BRD4 ChIP-seq was performed with NCI-H1963 SCLC cells treated either DMSO or dBET6 for 2 h. The representative track shows the occupancy of BRD4 at ASXL3 locus. **f** Whole-cell lysates were used for western blot with BRD2, BRD3, BRD4, Pol II, CDK9, and CCNT1 antibodies in human SCLC cell line NCI-H1963 treated with dBET6 for 0, 2, 8, and 24 h, *n* = 3. **g** Whole-cell lysates were used for western blot with ASXL3, JMJD6, NSD3, and CHD4 antibodies in human SCLC cell line NCI-H1963 treated with dBET6 for 0, 2, 8, and 24 h. HSP90 was used as an internal control, *n* = 3. **h** Whole-cell lysates were used for western blot with BRD2, BRD3, BRD4, ASXL3, JMJD6, NSD3, and CHD4 antibodies in human SCLC cell line NCI-H1963 treated with different concentrations of dBET6 for 8 h; HSP90 was used as an internal control, *n* = 3. **i** 8 different human SCLC cell lines NCI-H748, NCI-H1882, NCI-H1963, NCI-H209, NCI-H1436, NCI-H1105, NCI-H889, and NCI-H2171 were treated with different concentrations of dBET6 for 72 h. The cell viability was determined by CellTiter-Glo Luminescent Cell Viability Assay, *n* = 6. **j** The protein levels of ASXL3 and BRD4 from the previously cell lines mentioned (**i**) were determined by western blot, *n* = 3. **k** The representative track shows H3K27Ac and H3K4me1 levels at ASXL3 locus in ASXL3-high (NCI-H1963) and ASXL3-low (NCI-H2171) SCLC cells. **l** The Venn-diagram shows SE-associated genes in ASXL3-high and ASXL3-low cells from (**k**)

Based on the above results, loss of ASXL3 attenuates the proper function of BRD4 on chromatin. To determine the impact of ASXL3 on BRD4-dependent downstream genes, we treated SCLC cells with three different BET inhibitors, dBET6, JQ1, and IBET-151. The Log2 fold-change heatmap of metagene analysis showed that the gene expression patterns between ASXL3-depleted cells and BET inhibitor-treated cells were similar at non-TSS clusters (Fig. 5a). In total, there were 2417 genes significantly downregulated and 1743 genes significantly upregulated upon all of the three inhibitors treatment (Fig. 5b and S6A). Surprisingly, we found ASXL3 is a direct target of all three BET inhibitors (BETi), which resulted in the dramatic decrease in *ASXL3* gene expression (Fig. 5b and c). More specifically, we found that dBET6 is more efficient in repressing ASXL3 expression at the transcriptional level (Fig. 5c, Additional file 2: Figure S6B) in SCLC cells. To further confirm the RNA-seq data results, we treated NCI-H1963 SCLC cells with dBET6, JQ1, and IBET-151 for 4 h and 8 h and then determined the expression levels of *ASXL3* by real-time PCR. Consistent with the RNA-seq data, we found dBET6 treatment significantly reduced the mRNA levels of ASXL3, and another BRD4 target gene, *EIF4E*, within 4 h (Fig. 5d). To determine whether the regulation of ASXL3 by BET inhibitors were cell type specific, we treated another two SCLC cell lines, NCI-H748 and NCI-H1882, with dBET6, JQ1, and IBET-151 for 8 h. Consistent with our observations in NCI-H1963 SCLC cell lines, inhibition of BET proteins significantly reduced mRNA levels of ASXL3 in both cell lines (Additional file 2: Figure S6B). Mechanistically, we found 2-h treatment of dBET6 is sufficient to remove BRD4 occupancy from ASXL3 gene loci (Fig. 5e). To determine whether treatment of dBET6 could also reduce ASXL3 protein levels in SCLC cells, we treated NCI-H1963 SCLC cells with dBET6 in both dose- and time-dependent manners (Fig. 5f-h). A fast degradation of all BRD family proteins was observed (Fig. 5f and h). As expected, ASXL3 protein was reduced by dBET6 treatment in both dose- and time-dependent manners (Fig. 5g and h) and is also shown to be effective in other SCLC cell lines, such as NCI-H69, NCI-H1982, and NCI-H748 (Additional file 2: Figure S6C). Interestingly, we did not notice any significant changes among other BRD4-ET domain binding protein, such as JMJD6, CHD4, and NSD3 (Fig. 5g-h). According to previous studies, SCLC cells are more sensitive to BET inhibitors (BETi) compared to non-small cell lung cancer (NSCLC) cells [50]. However, there still remains a spectrum of BETi sensitivity within SCLC cells. Since ASXL3 is involved in the chromatin recruitment of BRD4 and maintenance of the BRD4-dependent gene expression, we sought to test whether ASXL3 expression levels are a determinant of sensitivity to BET inhibitor treatment. We selected eight different SCLC cell lines and treated the cells with different concentrations of dBET6 for 72 h. As a result, we found that SCLC cell lines NCI-H748, NCI-H1882, and NCI-H1963 were highly sensitive to dBET6 treatment, compared to more resistant SCLC cell lines, such as NCI-H889 and NCI-H2171 (Fig. 5i). Interestingly, the protein levels of ASXL3 in these cells were strongly correlated with drug sensitivity (Fig. 5j). However, we observed a similar trend but with less of a difference in cell viability between ASXL3-high and ASXL3-low SCLC cells with other BET inhibitors, such as JQ1 and iBET151 (Additional file 2: Figure S6D and S6E). These phenomena are not fully understood but could potentially be explained by differences in the effectiveness of BETi based on function. In addition, we directly showed the lethal effects of JQ1 and dBET6 at the same concentrations and compared the results to depletion of ASXL3 and BRD4 in the ASXL3 high (NCI-H1963) versus ASXL3 low (NCI-H2171) SCLC cells (Additional file 2: Figure S6G). Overall, our results suggest that ASXL3 could be used as an indicator for drug sensitivity for BET inhibitors, and dBET6 might be a better drug for SCLC treatment in clinical applications, especially for ASXL3-highly expressed SCLC patients. We further compared the super enhancer (SE)-associated genes between ASXL3-high (NCI-H1963) and ASXL3-low (NCI-H2171) SCLC cells, based on ranking H3K27Ac ChIP-seq signals. As a result, our findings indicated that ASXL3 is one of the 199 SE-associated genes in NCI-H1963 SCLC cells, but not in NCI-H2171 SCLC cells (Fig. 5k and l).

### Model-genetic interaction between BRD4 and PR-DUB.3

**Fig. 6:**
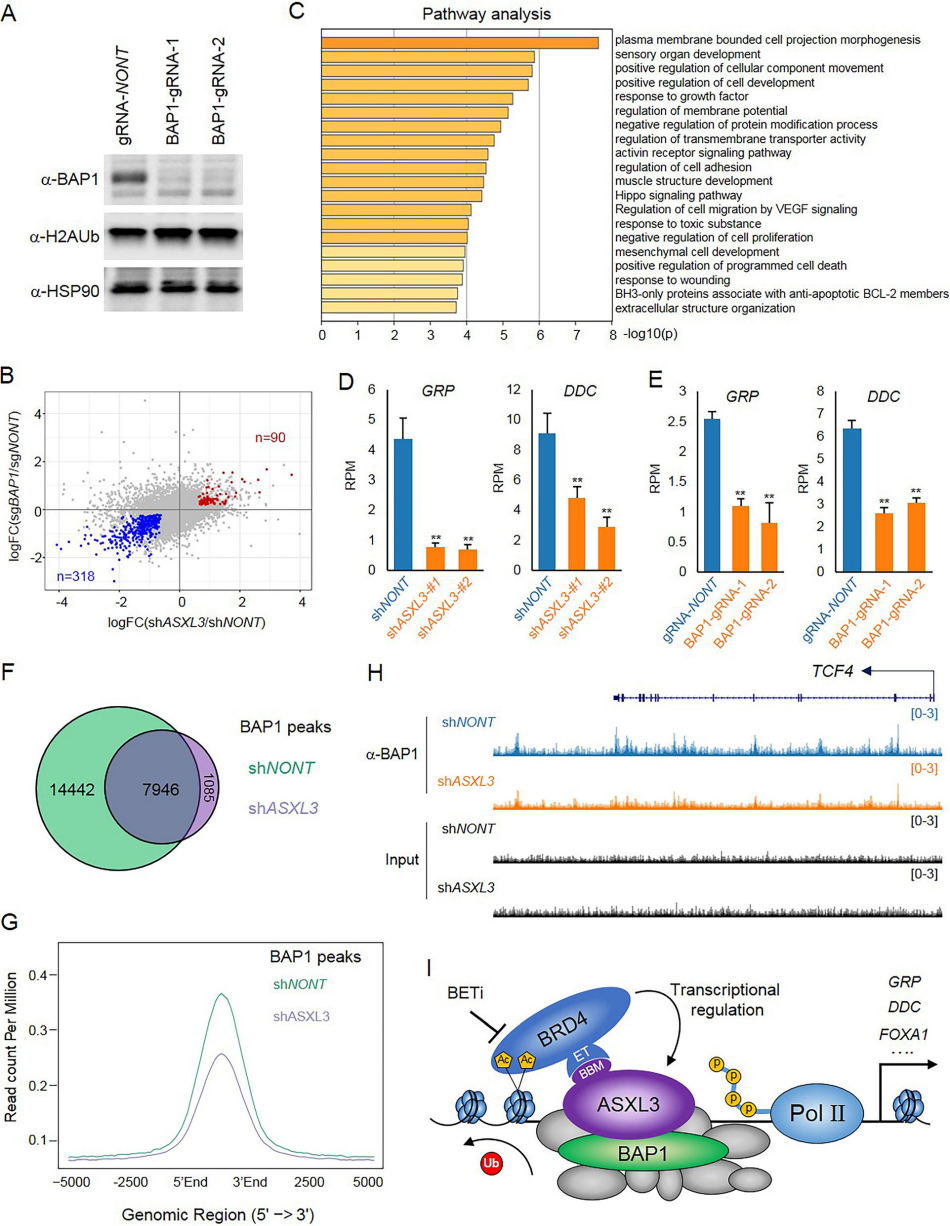
Model-genetic interaction between BRD4 and PR-DUB.3. **a** Whole-cell lysates were used for western blot with BAP1 and H2AK119Ub antibodies in human SCLC cell line NCI-H1963 transduced with either non-targeting CRISPR gRNA or BAP1 specific gRNA. HSP90 was used as an internal control, *n* = 3. **b** The scatter plot shows the overlap between ASXL3 and BAP1 targeted genes. **c** Pathway analysis by Metascape of genes that are positively correlated with both ASXL3 and BAP1 in NCI-H1963 cells. **d** The RNA-seq data for the expression of *GRP* and *DDC* genes in NCI-H1963 cells transduced with non-targeting shRNA or ASXL3-specific shRNAs, *n* = 2, two-tailed unpaired Student’s *t* test. **P < 0.01; *P < 0.05. **e** The RNA-seq data for the expression of *GRP* and *DDC* genes in NCI-H1963 SCLC cells transduced with non-targeting CRISPR gRNA or gRNA targeting *BAP1* gene, *n* = 2. The Venn-diagram (**f**) and average plot (**g**) shows BAP1 peaks and occupancy in NCI-H1963 cells transduced with either sh*NONT* or sh*ASXL3*. **h** Representative track example that shows BAP1 occupancy at enhancers between NCI-H1963 cells transduced with either sh*NONT* or sh*ASXL3*. **i** A model of targeting the positive feedback within BRD4/ASXL3/BAP1 axis for novel SCLC therapy

Since ASXL3 is an exclusive core subunit within BAP1 complex, to investigate whether BAP1 is involved in ASXL3-mediated cell identity, we depleted BAP1 by CRISPR/Cas9 with two different guide RNAs (Fig. 6a). Interestingly, by RNA-seq analysis, we found a striking overlap between BAP1 and ASXL3 target genes (Fig. 6b, Additional file 7: Table.S6). In total, there were 318 genes that are downregulated in both BAP1 and ASXL3-depleted cells and only 90 genes that are upregulated (Fig. 6b, Additional file 5: Table S4). Depletion of BAP1 also lead to a significant reduction in the expression of NE markers, such as *GRP* and *DDC* (Fig. 6b-d), indicating the critical function of BAP1/ASXL3 complex in maintaining proper function of NE small cell lung cancers. Finally, to investigate the mechanism as to how loss of ASXL3 can affect the function of BAP1 within SCLC cells, we performed BAP1 ChIP-seq in NCI-H1963 cells transduced with either non-targeting shRNA or ASXL3 shRNA. As a result, we detected 22,388 BAP1 peaks in ASXL3 wild-type cells and only 9031 BAP1 peaks in ASXL3-depleted cells (Fig. 6f). Consistent with the Venn-diagram analysis, the average plot also shows the reduction in BAP1 occupancy after ASXL3 depletion (Fig. 6g and h). These results suggest that similar to ASXL1 and ASXL2, ASXL3 is also responsible for BAP1 chromatin recruitment.

## Discussion

BRD4 is member of the bromodomain and extra-terminal (BET) family, which functions as a master transcriptional coactivator in both transcription and elongation, via a direct interaction with p-TEFb complex at its C-terminal domain [51, 52]. BRD4 could be recruited to chromatin through multiple mechanisms. Original studies have demonstrated that BRD4 could bind to di- and tetra-acetylated histone H4 and di-acetylated histone H3 tail via its bromodomain at the N-terminus [53, 54]. Emerging evidence has revealed that certain transcription factors and/or epigenetic factors have a major mechanistic role in recruiting and directing BRD4 to chromatin [55, 56]. Within all BRD family members (BRD2, BRD3, BRD4, and BRDT), the extra-terminal (ET) domain is located in the middle region. Previous studies by different groups have identified several ET-domain-specific binding proteins, such as JMJD6 [45], NSD3 [57], CHD4 [47], and MLV [58]. Interestingly, compared to other ET-domain binding factors, ASXL3 is a tissue-specific factor that is highly expressed in SCLC, and has not been identified before as a BRD4 binding protein. We found ASXL3 has a similar BRD4-binding motif (BBM) as other ET-domain binding proteins; however, the functions of BBM containing proteins are different. For instance, JMJD6 [45], an arginine demethylase, could be recruited by BRD4 to demethylate H4R3me2s, which is directly read by 7SK snRNA, and decapping/demethylation of 7SK snRNA, ensuring the dismissal of the 7SK snRNA/HEXIM inhibitory complex [59]. In human AML leukemia cells, the short form of NSD3 could be recruited by BRD4 to *Myc* enhancers and activate *Myc* expression. However, we found ASXL3 itself is a direct transcriptional target of BRD4, and loss of ASXL3 attenuates BRD4 occupancy and function at enhancers. The global co-function between ASXL3 and BRD4 indicated that a positive feedback between ASXL3 and BRD4 is essential for ASXL3-highly expressed SCLC viability. Interestingly, we also noticed there is a portion of novel enhancer binding sites of ASXL3 that are BRD4-independent, which may due to BRD4 ChIP-seq efficiency and ASXL3 may also have other functions that are independent from its association with BRD4.

Dysregulation or mutations within ASXL family are often observed in different human cancers such as AML, CMML [60], breast cancer [61], and lung cancer [62]. Gain-of-function (GOF) mutations within ASXL1 gene have been reported as drivers for leukemogenesis [63]. One possible mechanism is the truncated ASXL1 could interact with BRD4, leading to the openness of chromatin in leukemia cells. Consistent with their findings, we did not detect the interaction between BRD4 and endogenous full-length ASXL1 (Fig. 2a and b). Instead, we found ASXL3 is the only additional sex-combs-like protein that interacts with BRD4, and we further identified a specific BRD4 binding motif (BBM) within ASXL3 that directly mediates the binding. In addition, based on our ChIP-seq, we found more than 60% of ASXL3 peaks are localized at enhancer regions, which is quite different from ASXL1 protein, which instead binds to the promoter regions in cells [64]. As a result, ASXL3/BAP1 sub-complex may function as oncogenic protein complex in cells, especially in SCLCs. Thus, selective inhibition of ASXL3/BAP1 protein levels or activity may be a novel therapeutic strategy for SCLC treatment.

Previous studies have demonstrated that SCLC cells are more sensitive to BET inhibitor treatments, compared to non-small cell lung cancer (NSCLC) cells [50, 65]. However, we found not all of the SCLC cells have equal sensitivity to BET inhibitors. Here we demonstrated that ASXL3 is essential for BRD4 recruitment to chromatin and also responsible for the expression of BET protein-targeted genes. We hypothesize that the cells with high levels of ASXL3 expression might be more dependent on BET protein-targeted genes. Indeed, based on our RNA-seq data analysis, we have identified 142 genes downregulated in both ASXL3-depleted cells and different BETi-treated cells. It is noteworthy that a number of programmed cell death pathway genes were significantly enriched within the 142 gene list, such as *BCL2*, *BDNF*, *KIT*, and *HDAC9*. Therefore, ASXL3 could be used as an independent marker to predict the sensitivity towards BETi treatment. Moreover, other studies have provided evidence that further supports our results involving the direct interactions between BRD4 and ASXL3. For instance, pharmacologically induced inhibition of BET proteins’ activity in young mice could lead to Autism-like syndrome (ASD) [66], which is very similar to the phenotype seen in ASXL3 mutant (+/fs) patients [67]. In addition, it has been demonstrated that ASXL3 functions as a pluripotency factor in human small lung cancer [18]. As a result, the ASXL3/BRD4 axis may also contribute to the normal neurological development.

*ASCL1* gene encodes a member of the basic helix-loop-helix (BHLH) family of transcription factors. ASCL1 activates transcription by binding to a typical E box (5′-CANNTG-3′) DNA motif and is essential for pulmonary NE cells (PNECs) development [68] as well as SCLC cells viability [69]. In SCLC, *ASCL1* defines a subtype of SCLC (SCLC-A), with low expression of *NEUROD1*, *POU2F3*, and *YAP1* [38]. Due to *ASCL1* expression being significantly enriched in *ASXL3*-highly expressed cells, we sought to determine whether ASCL1 functions as a direct transcription factor of ASXL3. However, depletion of ASXL3 or ASCL1 does not affect the expression of one another. This result indicates that ASXL3 and ASCL1 might be controlled by similar upstream factors instead. Based on our RNA-seq data, depletion of ASXL3 dramatically reduced the expression of the NE markers, such as *GRP* and *DDC*; however, the expression of *NEUROD1*, *POU2F3*, and *YAP1* does not increase in ASXL3-depleted cells. These results suggest that ASXL3 may not be able to determine cell type; however, it is essential for maintaining the proper function and feature of SCLC-A type of cancers. As a result, identification of the upstream factor of ASCL1 and ASXL3 may also help to better understand the collaboration between ASXL3 and ASCL1 in SCLC. In summary, our studies have identified a BRD4/ASXL3/BAP1 epigenetic regulatory axis as a target for SCLC therapeutic treatment through BET inhibition (Fig. 6i).

## Conclusions

In this study, we have identified a critical link between Polycomb repressive deubiquitinase-BAP1 (PR-DUB) complex and BRD4, which is bridged by the physical interaction between additional sex combs-like protein 3 (ASXL3) in SCLC. We further show that the epigenetic machinery which is comprised of BAP1/ASXL3/BRD4 maintains the transcription activation at enhancers in SCLC. This study provides mechanistic insight into the oncogenic function of ASXL3 protein in SCLC, and suggests that ASXL3 could be used as a new biomarker for drug sensitivity for BET inhibitors. BET inhibitors/degraders might be moreeffective drugs for SCLC treatment in clinical applications, especially for ASXL3-high patients.

## Supplementary information

**Additional file 1: Table S1.** Primers used for real-time PCR and constructs.

**Additional file 2: Figure S1**. ASXL3 defines a subtype of SCLC with high expression of ASCL1. **Figure S2.** ASXL3 interacts with BRD4 in SCLC. **Figure S3**. Identification of direct BRD4 binding motif in ASXL3. **Figure S4**. ASXL3 is an enhancer binding factor in both human and mouse SCLC. **Figure S5.** Enhancer ASXL3 determines the expression of nearby genes in SCLC. **Figure S6.** ASXL3 is a direct target of BET inhibitors and predicts drug sensitivity.

**Additional file 3: Table S2.** Genes significantly enriched in the *ASXL3*-high and *ASXL3*-low expressed group of SCLC cells.

**Additional file 4: Table S3.** Mass spectrometry results for ASXL3 purification.

**Additional file 5: Table S4.** ASXL3 target genes that are occupied by ASXL3 at TSS or NonTSS regions.

**Additional file 6: Table S5.** Super enhancers that are co-occupied by both ASXL3 and BRD4.

**Additional file 7: Table S6.** Genes that are co-regulated by BAP1 and ASXL3.

## Data Availability

The RNA-seq data from 1004 human cell lines with refined tumor type annotations were obtained from Cancer Cell Line Encyclopedia Data Portal Ghandi et al. [30]. ATAC-seq data were downloaded from Gene Expression Omnibus (GEO) for NCI-H1963 (GSM3321013) and NCI-H2171 (GSM3321014) cell lines with accession number GSE118207 [70]. NGS data generated for this study are available at the Gene Expression Omnibus (GEO) under accession number GSE145028 [71]. The source code of Ceto pipeline used for analyzing the NGS data from this study is available at the Github site: https://github.com/ebartom/NGSbartom [72]. Mass spectrometry data in this study is included in this manuscript (Additional file 4).
