# Supplementary material for: ASXL3 bridges BRD4 to BAP1 complex and governs enhancer activity in small cell lung cancer
Source: Genome Med. 2020 Jul 15;12:63. doi: 10.1186/s13073-020-00760-3 (PMC7362484; doi:10.1186/s13073-020-00760-3)
Supplement: Supplementary file 2 — Additional file 2: Figure S1. ASXL3 defines a subtype of SCLC with high expression of ASCL1. Figure S2. ASXL3 interacts with BRD4 in SCLC. Figure S3. Identification of direct BRD4 binding motif in ASXL3. Figure S4. ASXL3 is an enhancer binding factor in both human and mouse SCLC. Figure S5. Enhancer ASXL3 determines the expression of nearby genes in SCLC. Figure S6. ASXL3 is a direct target of BET inhibitors and predicts drug sensitivity. [file 13073_2020_760_MOESM2_ESM.docx]

**Supplementary Figure**
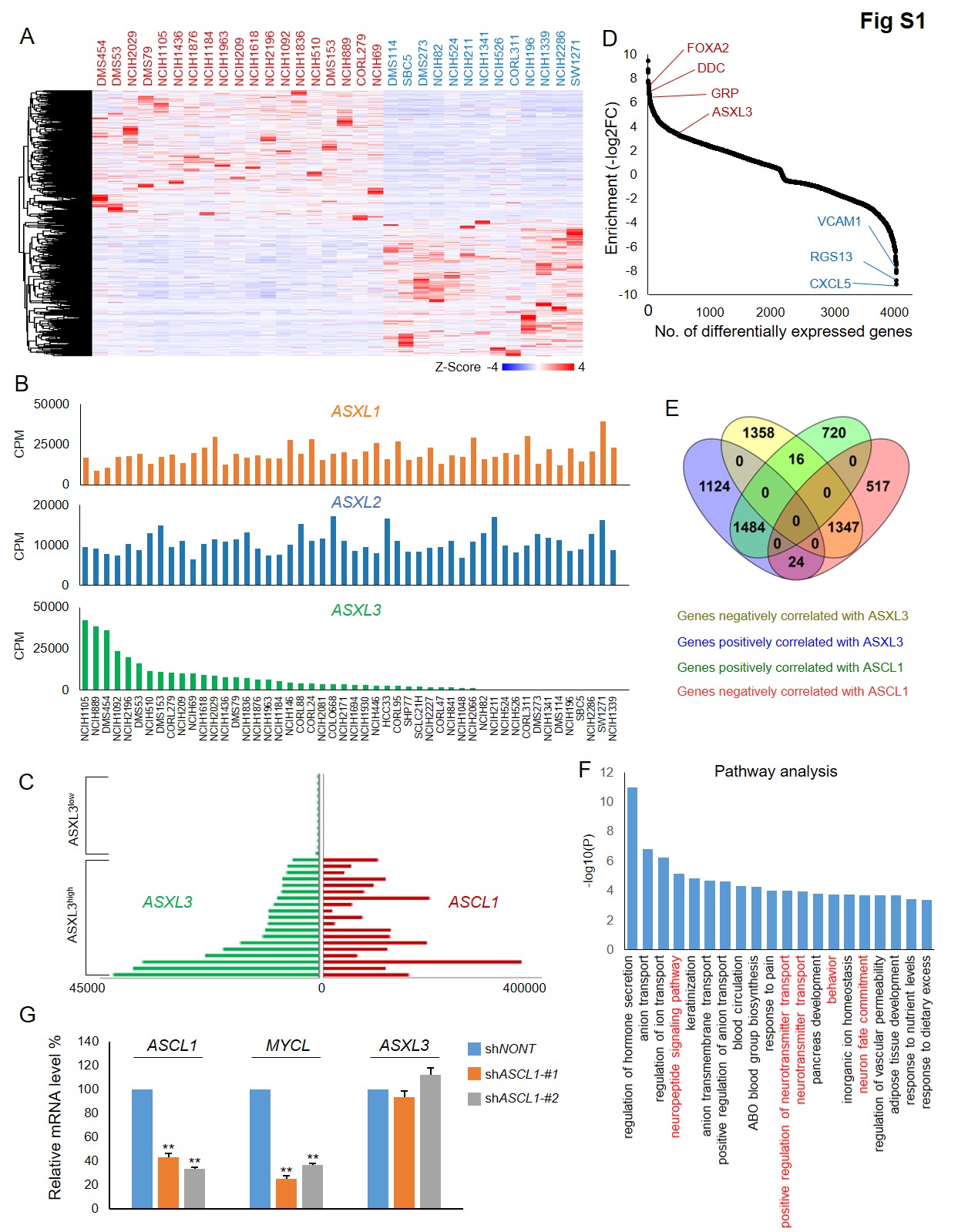


**Fig S1. ASXL3 defines a subtype of SCLC with high expression of ASCL1**

A) The heatmap shows the significant enriched transcripts in ASXL3-high and ASXL3-low groups of SCLC cell lines. B) The expression level (CPM value) of ASXL1, ASXL2, and ASXL3 across all 50 SCLC cell lines presented in (A). C) The expression level (CPM value) of ASXL3 and ASCL1 in ASXL3-high and ASXL3-low SCLC cells. D) The most significantly enriched genes that are positively correlated with ASCL1 expression in SCLC cells. E) The Venn-diagram shows the overlap of genes correlated with ASXL3 and/or ASCL1 in SCLC cell lines. F) Pathway analysis by Metascape of 1,484 genes that are positively correlated with both ASXL3 and ASCL1 in SCLC cell lines. G) The relative mRNA levels of ASCL1, MYCL, and ASXL3 in NCI-H1963 SCLC cells transduced with non-targeting shRNA or ASCL1 specific shRNAs, n=3.


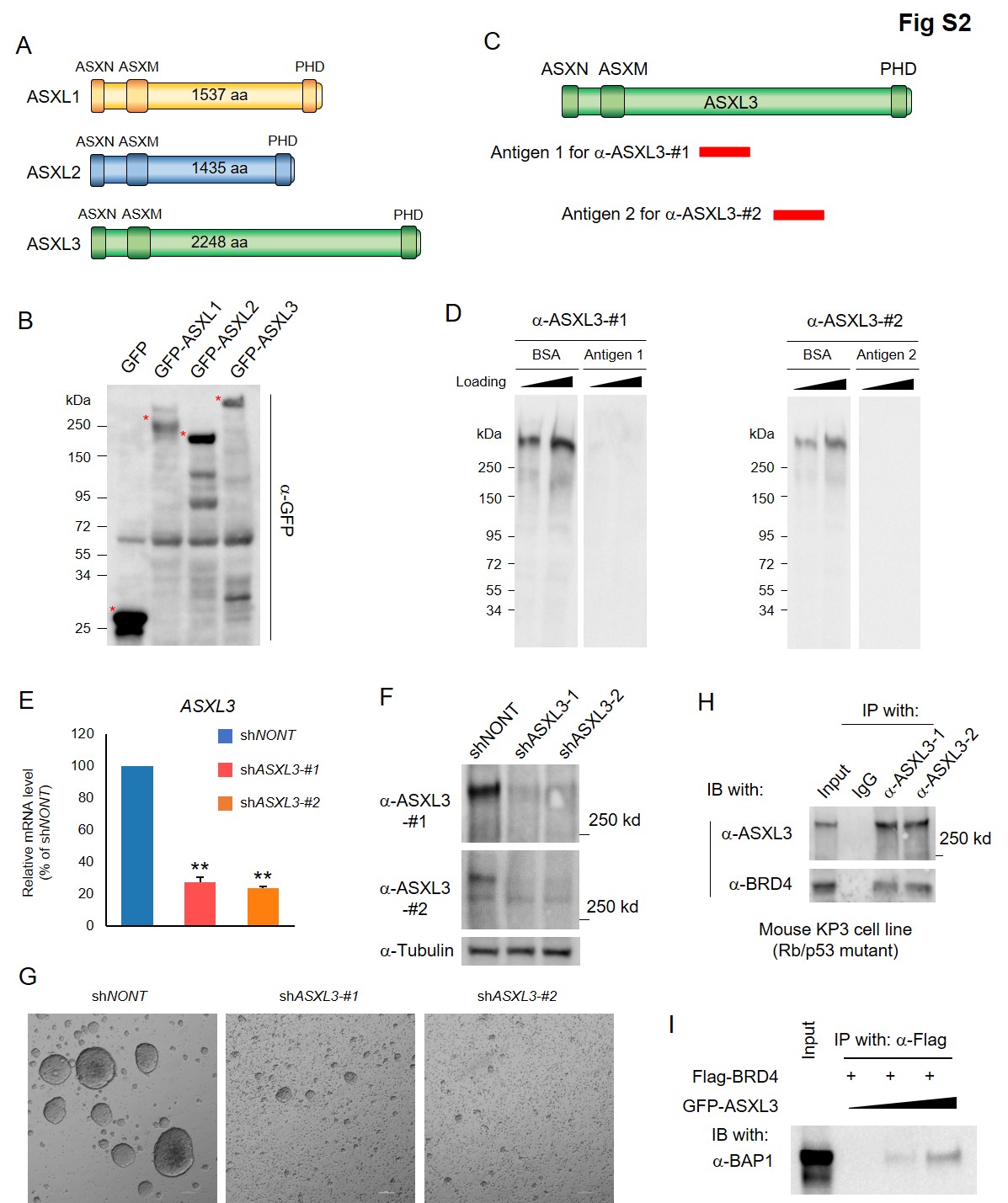


**Fig S2. ASXL3 interacts with BRD4 SCLC**

A) Structure of three additional sex-comb like proteins, ASXL1, ASXL2, and ASXL3.

B) ASXL1, ASXL2, and ASXL3 was expressed as a GFP-tagged fusion protein in HEK293T cells. C) Schematic of human ASXL3 protein and the antigen peptide for generation of the polyclonal antibodies. D) Peptide blocking assay was performed to validate specificity of each ASXL3 polyclonal antibody made in-house. Recombinant antigen peptides (final concentration 100 μg/ml) were used as blocking peptides, BSA (100 μg/ml) was used as a negative control. E) The relative mRNA levels of *ASXL3* was determined by real-time PCR in NCI-H1963 SCLC cells transduced with non-targeting shRNA or *ASXL3* specific shRNAs, n=3. F) The protein level of ASXL3 was determined by western blot in NCI-H1963 SCLC cells transduced with non-targeting shRNA or ASXL3 specific shRNAs, with two different ASXL3 antibodies made in-house, n=3. G) Representative photograph shows the colony formation in SCLC cells transduced with non-targeting shRNA or ASXL3 specific shRNAs. H) IP of endogenous ASXL3 of mouse SCLC cell line KP3 followed by IB for BRD4; IgG was used as negative control, n=3. I) HEK293T cells were co-transfected with plasmids expressing Flag-tagged BRD4, with a gradual increase in the amount of plasmid expressing GFP-tagged ASXL3 per experiment. Immunoprecipitation (IP) from whole cell lysates was performed with antibodies against the Flag epitope, followed by immunoblotting (IB) with antibodies against BAP1, n=3.


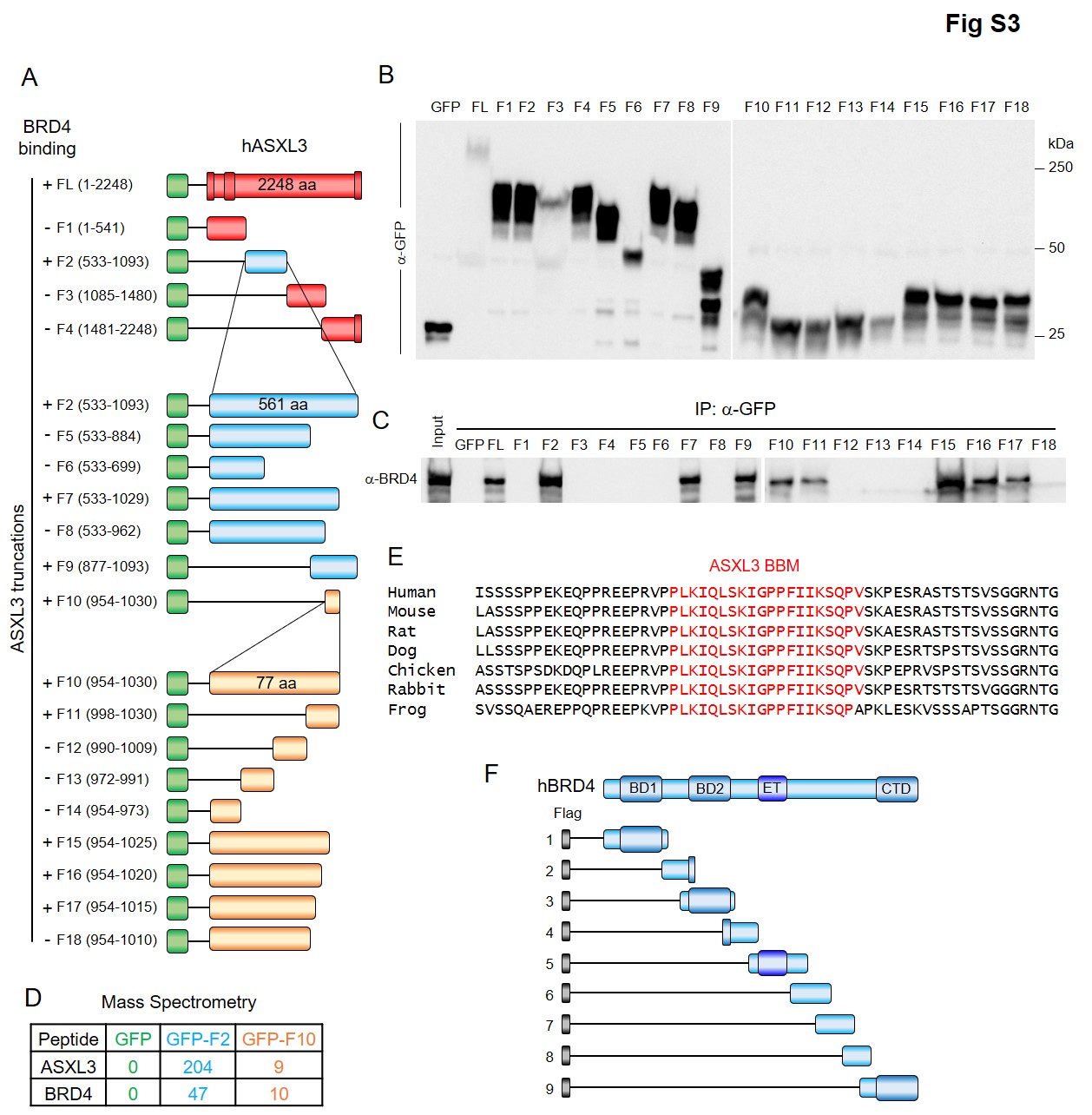


**Fig S3. Identification of direct BRD4 binding motif in ASXL3**

A) Schematic diagram depicting the domain organization of the human ASXL3 protein. The indicated fragments were sub-cloned as a GFP-tag fusion into pLNCX-GFP plasmid. B) Whole-cell lysates were used for western blot with GFP antibodies in cells transfected with empty vector (GFP) or different ASXL3 fragments in (A), n=3. C) Whole-cell lysates were used for immunoprecipitation (IP) with GFP antibodies followed by immunoblotting (IB) for BRD4 in cells transfected with empty vector (GFP) or ASXL3 fragments in (A), n=3. D) Fragment F2 and Fragment F10 of ASXL3 was expressed as a GFP-tagged fusion protein in HEK293T cells and subjected to GFP-purification from nuclear extracts and used for mass spectrometry analysis. Peptide numbers of ASXL3 as well as BRD4 purified by GFP-ASXLs were shown. E) CLUSTALW alignment analysis of vertebrates with ASXL3-BBM sequences. F) Schematic diagram depicting the domain organization of the human BRD4 protein. The indicated fragments were sub-cloned as a Flag-tagged fusion into pLNCX plasmids.


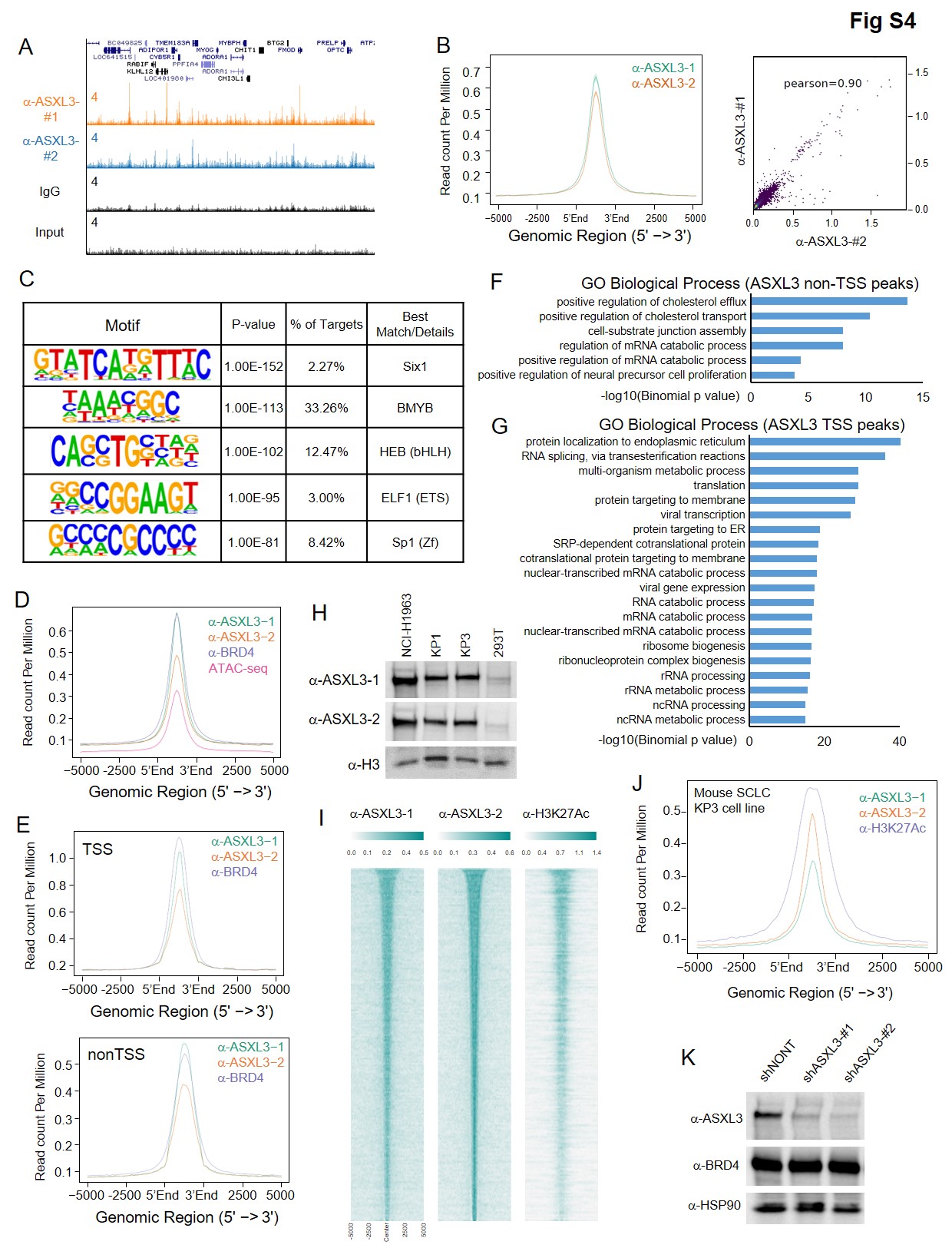


**Fig S4.** **ASXL3 is an enhancer binding factor in both human and mouse SCLC**

A) Representative track example that shows ASXL3 ChIP-seq with two different homemade antibodies; IgG was used as negative control, n=2. B) The average plot shows the co-localization of ASXL3 peaks detected by two antibodies (left panel), and the Pearson Correlation coefficient value was determined by using multiBigwigSummary and plotCorrelation functions from deepTools (right panel). C) Motif analysis with the common peaks detected by both ASXL3 antibodies. D) The average plot shows the co-localization of both ASXL3 and BRD4 at open chromatin loci (ATAC-seq). E) The total ASXL3 peaks were divided into TSS and non-TSS regions. The average plot shows the co-localization of both ASXL3 and BRD4 at TSS (upper panel) and non-TSS regions (lower panel). The GO term pathway analysis was performed with ASXL3 peaks at non-TSS (F) and TSS (G) regions in NCI-H1963 SCLC cell line. H) Whole-cell lysates were used for western blot analysis with ASXL3 and histone H3 antibodies in human NCI-H1963 SCLC cell line, mouse SCLC cell line KP1, KP3 (p53/Rb mutant) cells, and 293T cells, n=3. The heatmap (I) and average plot (J) shows the co-localization of both ASXL3 and H3K27Ac via peak signals in mouse KP3 cells. K) Whole-cell lysates were used for western blot with ASXL3 and BRD4 antibodies in NCI-H1963 SCLC cells transduced with ASXL3 shRNA or non-targeting shRNA. HSP90 was used as an internal control, n=3.


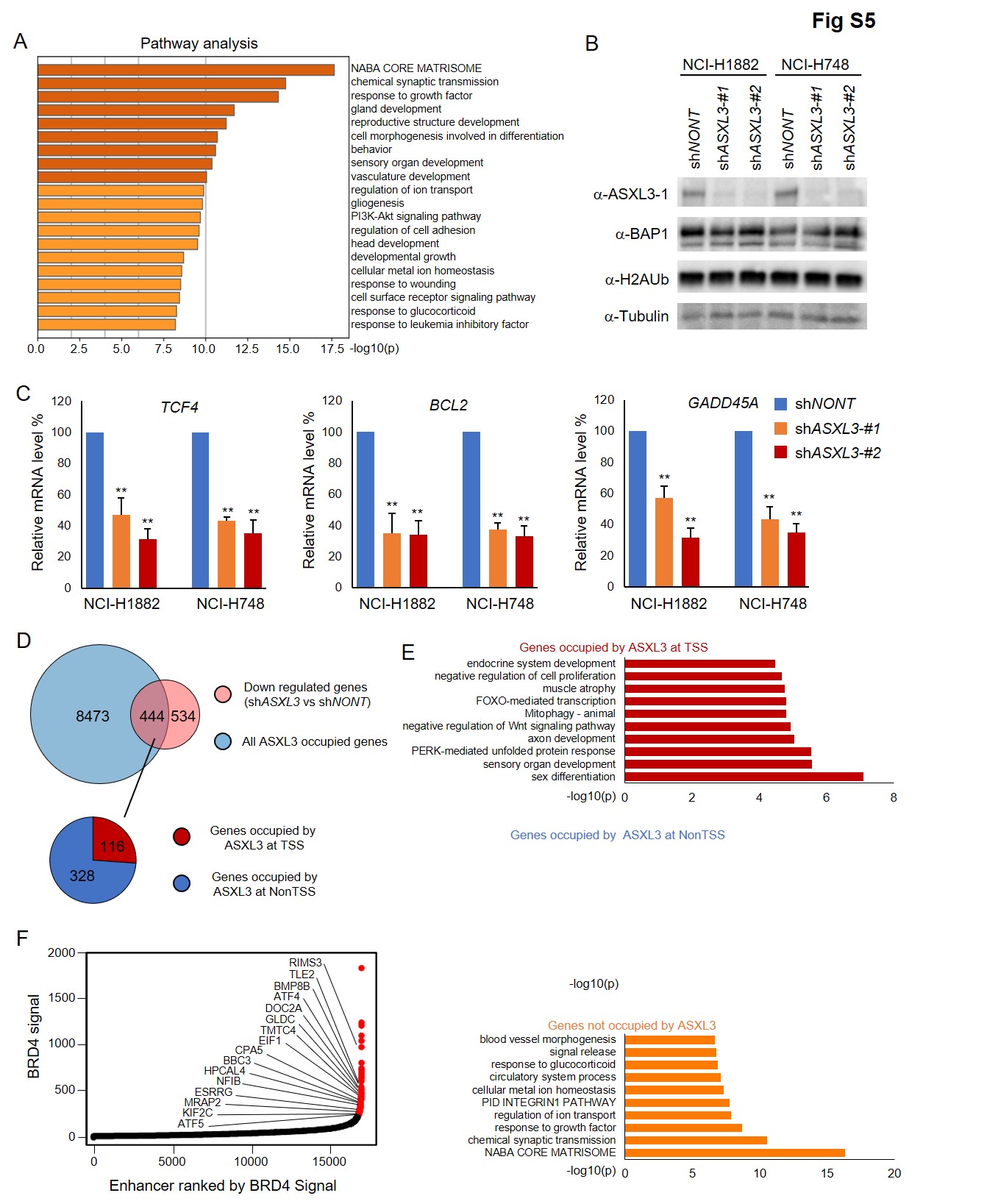


**Fig S5.** **Enhancer ASXL3 determines the expression of nearby genes in SCLC**

A) Pathway analysis by Metascape of the significantly down-regulated genes in both ASXL3-#1 and ASXL3-#2 shRNA treated cells, n=2. B) Whole-cell lysates were used for western blot analysis with ASXL3, BAP1, and histone H2AK119Ub antibodies in NCI-H1882 and NCI-H748 SCLC cells transduced with non-targeting shRNA or two different ASXL3 shRNAs, n=3. C) The relative mRNA levels of TCF4, BCL2, and GADD45A were determined by real-time PCR in NCI-H1882 and NCI-H748 SCLC cells transduced with non-targeting shRNA or two different ASXL3 shRNAs, n=3, two-tailed unpaired Student’s *t*-test. **P < 0.01; *P < 0.05. D) The Venn-diagram shows the overlap between the down-regulated genes in ASXL3-depleted cells and all ASXL3 occupied genes (upper panel). The pie plot at the lower panel shows the distribution of down-regulated genes occupied by ASXL3 at TSS or Non-TSS regions. E) The down-regulated genes in ASXL3-depleted cells are divided into three groups: genes occupied by ASXL3 at TSS regions; genes occupied by ASXL3 at non-TSS regions (NonTSS); and genes not occupied by ASXL3. Pathway analysis with each gene lists were determined by Metascape. F) ChIP-seq with BRD4 antibody identified putative super enhancers (SEs) in NCI-H1963 SCLC cells. Hockey-stick plot representing the normalized rank and signals of BRD4. Representative SE-associated genes that are controlled by ASXL3 are labeled.


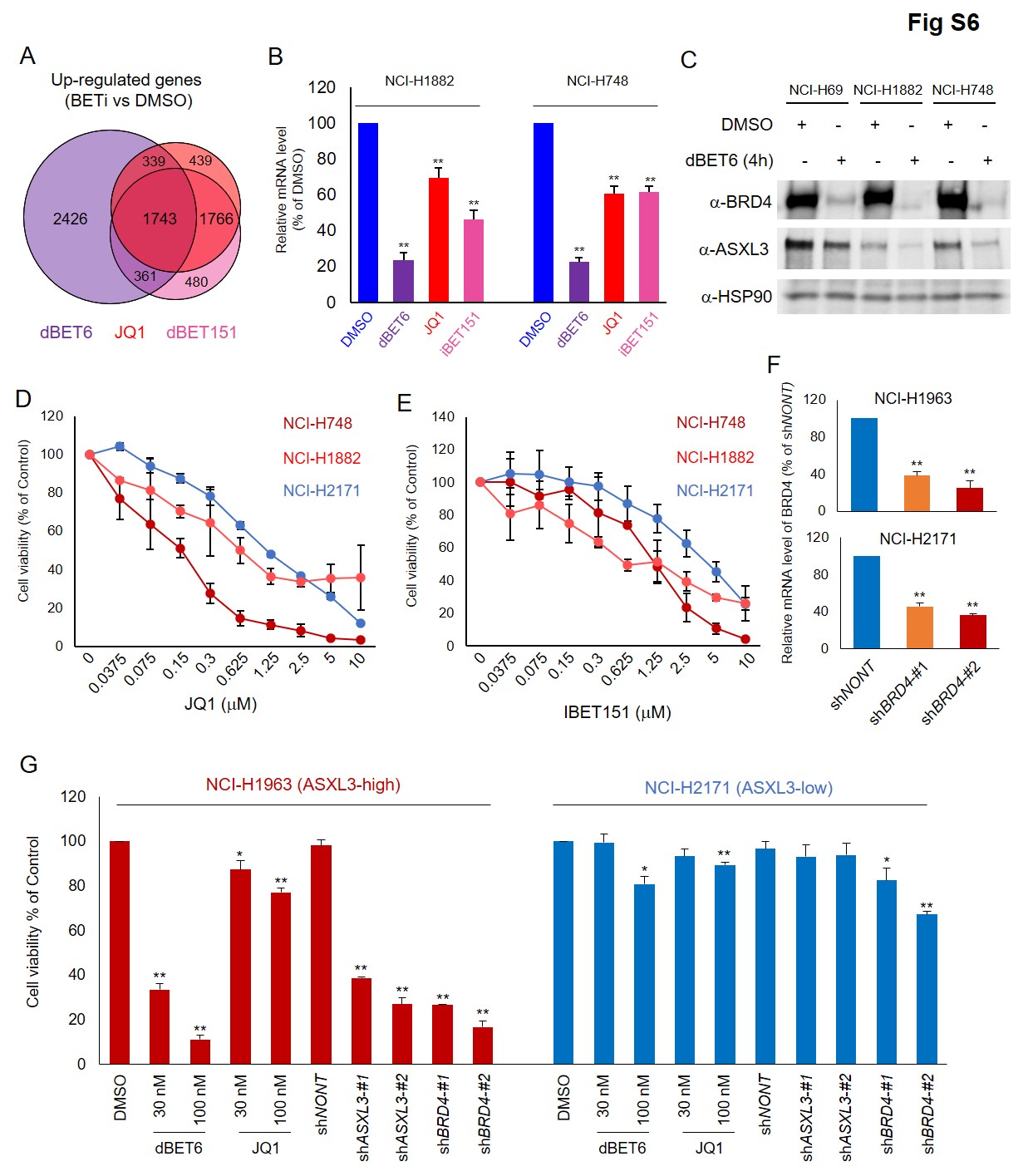


**Fig S6.** **ASXL3 is a direct target of BET inhibitors and predicts drug sensitivity**

A) The Venn-diagram shows the overlap of up-regulated genes in NCI-H1963 cells treated with dBET6 (300 nM), JQ1 (1 μM), or IBET-151 (1 μM), n=2. B) NCI-H1882 and NCI-H748 SCLC cells were treated with dBET6 (150 nM), JQ1 (1 μM), or IBET151 (1 μM) for 4 hours, the relative expression level of ASXL3 mRNA was determined by real-time PCR, n=3, two-tailed unpaired Student’s *t*-test. **P < 0.01; *P < 0.05. C) The protein levels of ASXL3 and BRD4 were determined by western blot analysis in NCI-H69, NCI-H1882, and NCI-H748 SCLC cells treated with either DMSO or dBET6 (150 nM) for 4 hours. HSP90 was used as an internal control, n=3. Three different human SCLC cell lines, NCI-H748, NCI-H1882 (ASXL3-high), and NCI-H2171 (ASXL3-low) cells, were treated with different concentrations of (D) JQ1 or (E) IBET151 for 72 hours. The cell viability was determined by CellTiter-Glo Luminescent Cell Viability Assay, n=6. F) The relative mRNA levels of *BRD4* was determined by real-time PCR in NCI-H1963 and NCI-H1963 SCLC cells transduced with non-targeting shRNA or *BRD4* specific shRNAs, n=3. G) Two different human SCLC cell lines, NCI-H1963 (ASXL3-high) and NCI-H2171 (ASXL3-low) were treated with 30 nM/100 nM of JQ1 or dBET6 for 72 hours. ASXL3 or BRD4 depleted cells were used as positive control. The cell viability was determined by CellTiter-Glo Luminescent Cell Viability Assay, n=6, two-tailed unpaired Student’s *t*-test. **P < 0.01; *P < 0.05.
